# Bioinspired Dual-Scale Crack Manipulation Enabling 325%-Stretchable Metal Film Conductors for AI-Empowered Electronic Skins

**DOI:** 10.1007/s40820-026-02152-5

**Published:** 2026-04-07

**Authors:** Tianming Sun, Bin Feng, Guisheng Zou, Jinpeng Huo, Bo Bi, Jin Peng, Zehua Li, Gongbo Bian, Bingang Xu, Lei Liu

**Affiliations:** 1 State Key Laboratory of Clean and Efficient Turbomachinery Power Equipment, Department of Mechanical Engineering, Tsinghua University, Beijing, 100084 People’s Republic of China; 2 Key Laboratory for Advanced Materials Processing Technology, Ministry of Education, Beijing, 100084 China; 3 Nanotechnology Center, School of Fashion and Textiles, The Hong Kong Polytechnic University, Hung Hom, Kowloon, 999077 Hong Kong People’s Republic of China; 4 Ningbo Institute of Materials Technology and Engineering, Chinese Academy of Sciences, Ningbo, 315201 People’s Republic of China

**Keywords:** Hierarchical, Artificial intelligence, Flexible sensors, Stretchable electronics, Stretchable conductors, Flexible conductors

## Abstract

The advancement of electronic skins toward commercialization calls for cost-effective and robust flexible conductors, among which metal films are attractive candidates but suffer from poor stretchability. Conventionally, cracks formed in strained metal films are universally considered detrimental and sought to be suppressed. Herein, we demonstrate that cracking is not a drawback, but rather an effective design parameter for on-demand tailoring of film properties. By exploiting the size effect in crack engineering, we propose a bioinspired, dual-scale architecture that endows metal film conductors with up to 325% stretchability. We show that through the synergy of nanoscale pore implantation and microscale substrate roughening, progressively evolved crack patterns can be programmed within metal films by modulating structural parameters. This tunable film cracking behavior yields a nearly 25-fold regulation of stretchability, representing a remarkably wide tunable range among previously deposited metal counterparts. Leveraging these advances, a stretchable metal film-based, AI-empowered electronic skin is constructed, demonstrating excellent practicality across diverse flexible sensing scenarios. Our strategy highlights the pivotal role of the size effect in precise crack manipulation, and the multi-scale regulation paradigm establishes a fresh route to performance improvement of flexible conductors.

**Supplementary Information:**

The online version contains supplementary material available at 10.1007/s40820-026-02152-5.

## Highlights

- A leaf-inspired hierarchical architecture is developed to enable micro-nano, dual-scale crack manipulation in metal films, leading to a nearly 25-fold regulation of stretchability (from 12% to 325%).
- A stretchable metal film-based, AI-empowered electronic skin is developed, demonstrating excellent applicability in human motion monitoring, sign language translation, and speech recognition.
- The role of metal film cracking in stretchable electronics is reinterpreted, shifting from an unfavorable failure phenomenon into a powerful design tool for on-demand tuning of electromechanical properties.

**Supplementary Information:**

The online version contains supplementary material available at 10.1007/s40820-026-02152-5.

## Graphical abstract

**Figure:**
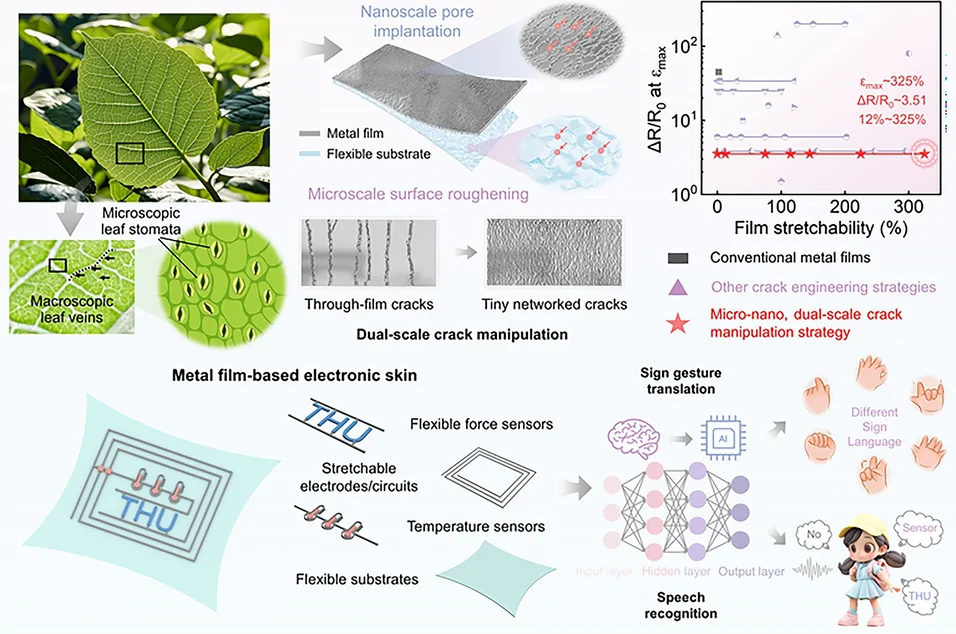

## Introduction

Mimicking the characteristics of human skin to perceive real-world information, electronic skins are ushering in a new era for the Internet of Things [1–10]. Thin-film flexible conductors, such as metal films [11], metal nanowire networks [12], and sintered metal nanoparticle films [13, 14], can serve as either flexible electrodes/circuits or force sensors, which are indispensable components for electronic skins. Currently, electronic skins are at a critical tipping point toward commercialization, which drives the need for cost-effective and robust materials suitable for scalable production [15–18]. Against this background, metal films are ideal candidates for flexible conductors due to excellent physical properties, abundant resources, and well-established manufacturing processes [19, 20]. These merits have established metal films as backbone conductors in conventional rigid electronics [21, 22]. However, their application in flexible electronics is constrained by intrinsically low stretchability [23, 24]. Therefore, developing ultra-stretchable metal films is a crucial step toward industrialization of stretchable electronic skins [25].

The poor stretchability of metal films primarily arises from the formation of through-film cracks under small strain [26, 27]. For decades, film cracking has been widely regarded as detrimental to electromechanical performance. Considerable efforts have been devoted to suppressing this unfavorable phenomenon via film wrinkling [28, 29], serpentine structure [30], interlayer regulation [31, 32], and two-stage film cracking [33]. Despite these beneficial advancements, the role of cracking in metal films warrants a re-examination. Because the electromechanical performance of metal films is governed by the film cracking behavior, a paradigm shift from simple “crack suppression” to intentional “crack manipulation” could open pathways to flexible conductors with broadly tunable and even on-demand performance.

Fundamentally, crack manipulation entails controlling the spatial characteristics of cracks—such as density, length, and width—in thin films [31, 33]. This inherently spatial character implies that size effects provide a fresh and powerful tool for the localized modulation of cracking behaviors [34, 35]. Moreover, implementing crack manipulation across multiple scales (e.g., coupling micro- and nano-level designs) is expected to enrich film cracking behavior. The multi-scale methodology has the potential to synergistically improve the overall electromechanical performance of thin films. To date, such a design perspective regarding size effect for crack manipulation remains largely unexplored.

Nature provides a wealth of biological species serving as invaluable inspiration for advanced structural designs in electronic skins [36–38], as exemplified by scorpion-inspired ultrasensitive strain sensors [39], pufferfish-inspired ultra-stretchable electrodes [32], and nacre-inspired biphasic electronic skins [6]. Plant leaves, in particularly, exhibit an exquisite hierarchical architecture. Macroscopic veins serve as load-bearing frameworks that redistribute mechanical stress and prevent catastrophic tearing, while microscopic stomata act as compliant micro-features that locally release stress and suppress crack propagation [40, 41]. This dual-scale synergy enables leaves to tolerate complex deformation and maintain structural integrity. This natural wisdom offers a key insight that the film electromechanical performance may be optimized via multi-scale crack manipulation, inspired by the hierarchical strain-management strategy observed in leaves.

In this work, we proposed a leaf structure-inspired hierarchical design that enabled micro-nano, dual-scale crack manipulation, which achieved metal-film conductors with up to 325% stretchability. We clearly reveal that the interplay between nanoscale pore implantation and microscale substrate roughening is synergistic for the stretchability improvement of metal films. By fine-tuning the structural parameters of this hierarchical system, gradually evolved crack patterns can be induced within metal films, which contributes to a 25-fold stretchability regulation (from 12% to 325%). This versatile strategy transforms common metal films such as Ag, Cu, Pd, and Ag–Cu alloy into highly stretchable conductors, suitable for use as wide-range force sensors or strain-insensitive flexible electrodes. Capitalizing on these advantages, an AI-empowered, metal film-based electronic skin was constructed and exhibited an excellent practicality across diverse flexible scenarios.

## Experimental Section

### Materials

Metal targets (Ag, Cu, Pd, Ag–Cu alloy; purity > 99.99%) and commercial sandpapers were obtained by Zhongnuo New Materials and Eagle Brand, respectively. The microbump-roughened polydimethylsiloxane (PDMS) substrates were prepared as shown in Fig. S1. First, the silicone elastomer base and curing agent (SYLGARD™ 184, Dow Corning) were mixed at a mass ratio of 10:1 and degassed under vacuum for 30 min. The degassed mixture was then poured onto the templated sandpaper with different grits (400#, 800#, 4000#). After thermal curing at 90 °C for 6 h, the microstructures (i.e., microbumps) on the sandpaper surface were faithfully replicated on the PDMS substrate.

### Fabrication of the Proposed Dual-Scale Crack-Manipulated Metal Film Conductors

**Fig. 1:**
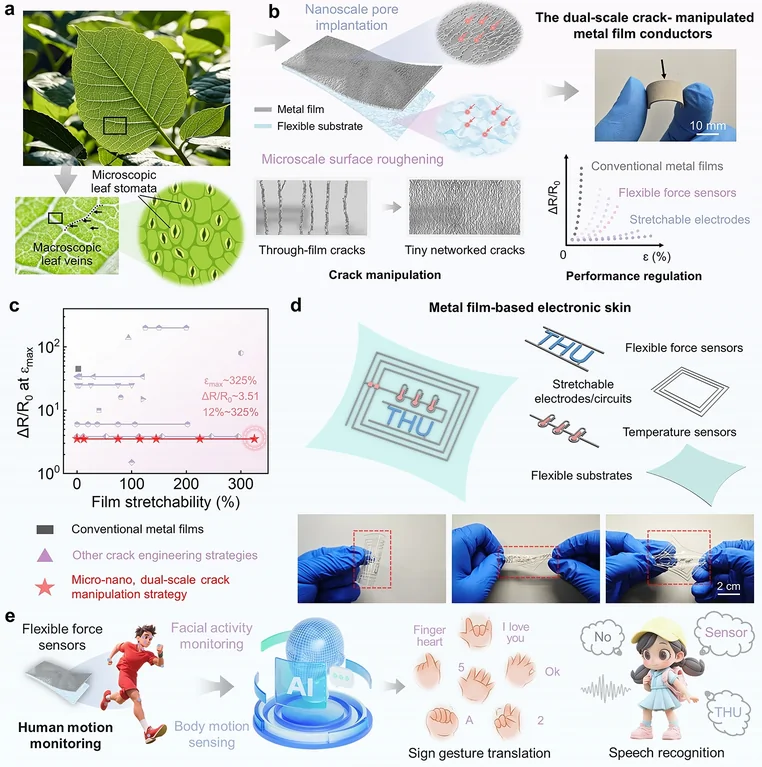
Design concept and overall performance of leaf-inspired metal film conductors based on the micro-nano, dual-scale crack manipulation strategy. **a** Schematic of a leaf structure. **b** Leaf-inspired hierarchical design of dual-scale crack-manipulated metal film conductors. Upper panel: bioinspired architecture and structure of the proposed metal film. Lower panel: evolution of film cracking behavior and corresponding electromechanical performance regulation. **c** Performance comparison between the proposed dual-scale crack-manipulated metal film and previously deposited metal counterparts in terms of stretchability, relative resistance change at the maximum stretchability, and the tunable range of stretchability [11, 23, 32, 34, 35, 43–49]. **d** Schematic of an all-metal-film-based electronic skin integrating diverse components. Lower subfigures: flexibility of the as-fabricated electronic skin. **e** Left panel: application prospects of metal film-based flexible sensors in human motion monitoring; Right panel: AI-empowered flexible sensors for sign gesture translation and speech recognition

Conventional or nanopore-implanted metal films were directly deposited onto smooth or surface-roughened substrates using a pulsed laser deposition (PLD) system comprising a commercial ultrafast laser (Edgewave PX100-2-GH, wavelength: 1064 nm, pulse duration: 10 ps), an optical path module, and a deposition chamber. The high-energy beam was directed through mirrors (Thorlabs) and a scanning galvanometer (SCANlab, IntelliCube 14) and focused onto the target to ablate material in vacuum or Ar atmosphere. During deposition, the laser power and pulse frequency were fixed to 60 W and 300 kHz, respectively. The various nanopore-implanted metal films were fabricated by regulating the chamber pressure during the PLD process (0.5, 300, and 1000 Pa, Note S1). To accurately evaluate electromechanical performance, Ag wires were attached to both ends of the deposited metal film as external electrodes, and subsequently the assembly was encapsulated by PDMS molding. For the all-metal-film-based electronic skin shown in Figs. 1d and 3c (later), different Ag films (conventional or nanopore-implanted) were selectively deposited on predefined regions of the PDMS substrate using a patterned mask.

### Material Characterization and Sensing Performance Measurement

The surface morphology and composition of the as-fabricated metal films were characterized by a scanning electron microscope (SEM, ZEISS Gemini 300) equipped with an energy-dispersive X-ray spectrometer (EDS, Xplore30) and an optical microscope (OM, Olympus, DP72). Sandpaper morphology was characterized by a confocal laser scanning microscope (CLSM, ZEISS LSM 900). All electromechanical performance measurements were conducted on an electronic universal testing machine (SHIMADZU, EZ-LX HS) coupled to a Keysight B2911A precision source/measure unit. To demonstrate deformation tolerance, the proposed stretchable electrode/circuits were connected in series with an LED or a smartphone to form closed circuits. For the facial activity monitoring and body motion sensing in Fig. 4a (later), the sensors were placed on different parts of the human body (eyebrow, eye, cheek, neck, throat, finger, elbow, wrist, knee, and ankle). For sign gesture translation and speech recognition, the sensors were mounted on the five fingers and throat of the volunteer to record real-time signals during hand gestures or vocalizations. All volunteers participating in the study provided informed consent. Finite element simulations (ABAQUS) were performed to resolve the stress and deformation fields of microbump-roughened PDMS substrates and Ag films (Note S2).

### Deep Learning-Assisted Sign Gesture Translation and Speech Recognition

The CNN models were implemented in the PyTorch framework and used for both training and inference. For sign language translation, the input and output layers of the CNN model contained 5 and 8 neurons, respectively. For speech recognition, the input and output layers of the CNN model involved 14 and 3 neurons, respectively. For the proposed 8 gestures and 3 words/phrases, 480 sets of data and 180 sets of data were separately collected, of which 75% were used for training and 25% for testing. Training employed the Adam optimizer with mean-squared-error loss.

## Results and Discussion

### Design Concept of the Micro-Nano, Dual-Scale Crack Manipulation Strategy

The design concept of leaf-inspired, stretchable metal film conductors is schematically illustrated in Fig. 1. In natural leaves, macroscopic veins and microscopic stomata play key roles in strain management by redistributing mechanical loads and locally releasing stress, respectively (Fig. 1a) [40, 41]. Inspired by this biological hierarchy, we developed a micro-nano, dual-scale architecture. In this design, microscale surface structures (i.e., microbumps) act like leaf veins for stress redistribution, while nanoscale pore implantation within metal films mimics stomata by enabling localized stress relaxation (Fig. 1b). By tuning the structural parameters of this hierarchical architecture, a progression of crack patterns is induced in strained metal films. Consequently, common metal films, traditionally limited by poor stretchability, are endowed with widely tunable electromechanical performance, enabling their use as diverse stretchable components in electronic skins.

As a proof of concept, a nanopore-implanted Ag film was fabricated on a microbump-roughened PDMS substrate via a pressure-regulated laser deposition method (Figs. 1b and S2, S3). The resulting Ag film exhibits a stretchability of up to 325%, far exceeding the ~ 10% of typical conventional counterparts (gray data in Fig. 1c) [23]. The as-fabricated metal films show excellent strain-insensitive electrical behavior (Δ*R/R*_0_ = 3.51 at 325% strain), with a quality factor calculated to be 0.93 (*Q* value, defined as the ratio of strain to relative resistance change [42]). This remarkable electrical stability under large deformation confirms their high suitability as stretchable electrodes and circuits. Recently, several representative works managed to improve the film stretchability (purple dataset in Fig. 1c) [11, 32, 34, 35, 43–49]. Different from these efforts focusing on “crack suppression,” our strategy places emphasis on “crack manipulation” to enable rich and tunable crack patterns in metal films. Consequently, our strategy achieves a 25-fold stretchability regulation (from 12% to 325%), which represents a significantly wide tunable range compared with previously deposited metal counterparts (red star data in Fig. 1c and Table S1). Notably, the performance of these dual-scale crack-manipulated metal films is even comparable to that of other advanced stretchable materials, such as liquid metals, conductive polymers, and nanocomposite-based conductors (Table S2). These advancements effectively overcome the intrinsic poor stretchability of conventional metal films.

The resulting widely tunable stretchability enables the fabrication of metal films with on-demand electromechanical performance, making them suitable for diverse electronic skin applications. Leveraging this merit, an all-metal-film-based electronic skin that integrates flexible force sensors, temperature sensors, and stretchable electrodes/circuits was constructed (Fig. 1d). This device was achieved by depositing Ag film patterns with tailored electromechanical performance onto a PDMS substrate through a mask-deposition process. The resulting electronic skin maintains excellent structural integrity under complex deformations such as bending, twisting, and stretching, demonstrating outstanding mechanical robustness. Furthermore, the application potential of the metal film-based electronic skin was explored in the sensing of human motions (Fig. 1e). To enhance functionality, artificial intelligence (AI) was further incorporated to process the obtained sensing signals, allowing for the recognition of user gestures and the identification of spoken words. These capabilities highlight the potential of this technology for advanced medical services and personalized health monitoring.

### Micro-Nano, Dual-Scale Manipulation of Film Cracking Behavior and Underlying Mechanism

**Fig. 2:**
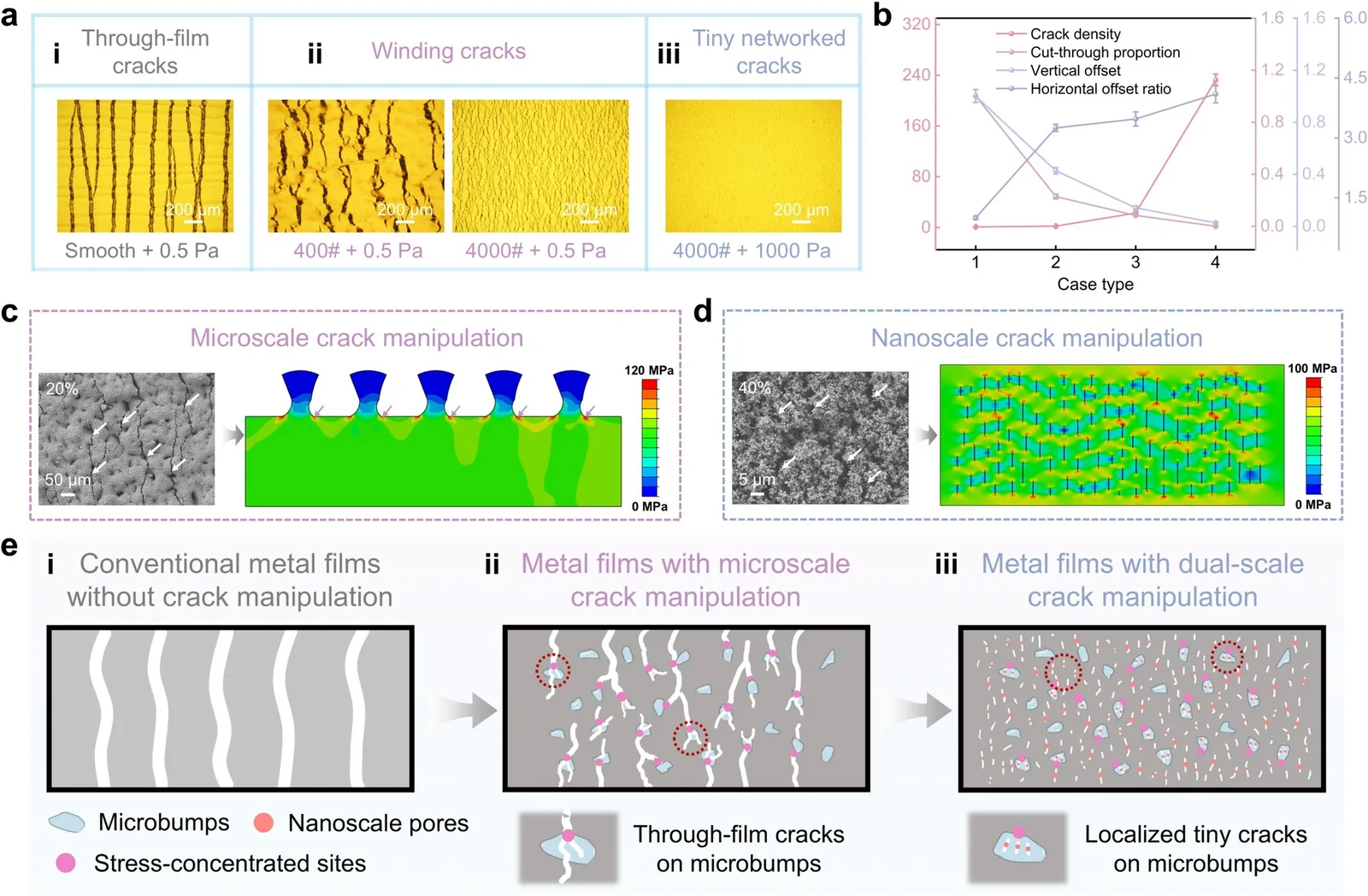
Cracking behaviors and regulation mechanisms of the dual-scale crack-manipulated metal film conductors. **a** Optical images showing three representative crack patterns engineered by the dual-scale crack manipulation: (i) through-film cracks; (ii) winding cracks; (iii) tiny-networked cracks. **b** Four normalized parameters (crack density, cut-through proportion, vertical offset, horizontal/vertical offset ratio) to characterize the features of various crack patterns in **a**. **c** Microscale crack manipulation: simulated stress distribution in compact metal films on microbump-roughened substrates under 20% strain. **d** Nanoscale crack manipulation: simulated stress distribution in the nanopore-implanted metal films under 40% strain. **e** Schematic illustration of the micro-nano, dual-scale crack manipulation strategy

The surface morphology of metal film conductors under different tensile strains based on the micro-nano, dual-scale hierarchical design was investigated. As shown in Figs. 2a-(i) and S4, metal films deposited on smooth substrates at low pressure (0.5 Pa) commonly exhibit through-film cracking behavior at small strain (< 20%), which is similar to previous works [23, 24]. This crack morphology easily leads to catastrophic electrical failure due to disrupted electron transport pathways (Fig. S5). In our design, microscale roughening of the substrate surface was firstly employed to regulate the film cracking behavior. Specifically, commercial sandpapers with abundant microbumps were selected as special templates for structuring the PDMS substrate and the molded microstructures were faithfully replicated in reverse (Fig. S1). Consequently, metal films located on the microbump-roughened substrate exhibit winding cracks instead of through-film cracks (Figs. 2a-(ii) and S6). Moreover, the morphological features of this crack pattern, such as crack density, length and width, can be adjusted by varying the microscale morphology (the distribution density of the microbumps or surface roughness) of substrate surface molded using sandpapers of different grit sizes (400# to 4000#, Fig. S7).

Further, nanoscale pore implantation was introduced to cooperatively regulate film cracking behavior. It is worth noting that the nanopore density within the metal films is highly associated with the deposition pressure (Note S1). By raising the chamber pressure during deposition, numerous nanopores emerge within the deposited metal film, transforming it from a compact to a loose structure (Fig. S8). Consequently, for the metal film deposited on the microbump-roughened substrate (sandpaper grit: 4000#), a high deposition pressure (300 Pa) drives winding cracks to evolve into networked cracks (Fig. S9). When the pressure was further increased to 1000 Pa, no obvious cracks were observed even at 80% strain (Figs. 2a-(iii) and S8). Instead, high-magnification images revealed a network of finer microcracks (Fig. S10). Compared with nanopore-implanted metal films without microscale substrate roughening, this dual-scale crack manipulation strategy enables better control over cracking, particularly on suppressing through-film cracks (Fig. S11). Moreover, the tiny‑networked crack pattern demonstrates favorable morphological reversibility during stretching and release (Fig. S12). To more quantitatively analyze the crack manipulation strategies, four normalized parameters, such as crack density, cut-through proportion, vertical offset, and horizontal/vertical offset ratio (Fig. S13 and Note S3), are defined to evaluate the crack pattern characteristics of each case in Fig. 2a. The key results are summarized in Fig. 2b. First, as the crack pattern evolves from through-film cracks to networked cracks, the crack density significantly increases owing to the emergence of many tiny cracks. Second, in the latter two crack patterns, the cracks no longer traverse the entire film, leading to a sharp drop in cut-through proportion. Finally, the vertical offset decreases, whereas the horizontal/vertical offset ratio increases. This implies that the long and straight cracks are progressively evolving into short and curved cracks, which corresponds to the emergence of winding and networked features. These results demonstrate rich crack pattern with diverse characteristics induced by the proposed strategy. We further demonstrated that this strategy is applicable to common metal films (Ag, Cu, Pd, and Ag–Cu alloy) to induce the transition of the cracking behavior from through-film cracks to tiny-networked cracks (Fig. S14).

More investigations were conducted to elucidate the underlying mechanisms of the proposed dual-scale crack manipulation by combining finite element analysis (FEA) with experimental observations. In our strategy, the incorporation of microscale surface structures (i.e., microbumps) contributes to stress redistribution during strain. FEA results reveal significant local stress concentrations at the base of microbumps (Fig. 2c), which are more susceptible to crack initiation during stretching. These stress-concentration sites promote the formation of a high-density, short cracks in strained metal films, which rapidly release strain energy and prevent the formation of long, straight through-film cracks (Fig. 2a-(ii) vs. 2a-(i)). Another consequence of the inhomogeneous stress distribution is that the tops of the microbumps experience minimal strain, resulting in delayed crack formation in these regions. This transition in cracking behavior allows the metal film to maintain essential electron transport pathways even under large strain, thereby effectively enhancing its stretchability (Fig. S15). However, as the overall nominal strain increases further, the local strain at the microbump tops also rises gradually (Fig. S16). Once the local strain exceeds the critical threshold for crack initiation—typically below 20% in metal films—cracks inevitably form even in these delayed-deformed regions. This phenomenon hints that at sufficiently large strain, extra crack manipulation should be incorporated beyond the substrate roughening strategy alone. Therefore, besides extrinsic microscale modification of the substrate surface, another intrinsic nanoscale modification of film properties is introduced. As shown in Figs. 2d and S17, nanopores implanted in metal films serve as sites for stress concentration on a more localized scale. Consequently, tiny-networked cracks can form in the metal films even on smooth substrates (Fig. S11), which preserves electrical conductivity by maintaining conductive pathways.

The dual-scale cooperative mechanism is summarized in Fig. 2e. First, the traditional compact metal films deposited on smooth substrates tend to form long and through-film cracks (Figs. 2e-(i) and S18). Second, the incorporation of microscale substrate roughening strategy promotes dense, short cracks due to microbump-induced stress inhomogeneity, thereby improving conductivity under large strain (Fig. 2e-(ii)). Finally, nanoscale film porosity modifications are further introduced to intrinsically alter cracking behavior by promoting more localized stress concentration. When the strain on the microbump tops (delayed-deformed regions) exceeds the cracking threshold, tiny-networked cracks form, effectively maintaining electrical pathways even under ultrahigh strain (Figs. 2e-(iii) and S19). This synergistic micro-nano, dual-scale crack manipulation ultimately leads to a significant improvement in film stretchability.

### Electromechanical Performance of the Proposed Crack-Manipulated Metal Films and Their Applications as Versatile Flexible Components

**Fig. 3:**
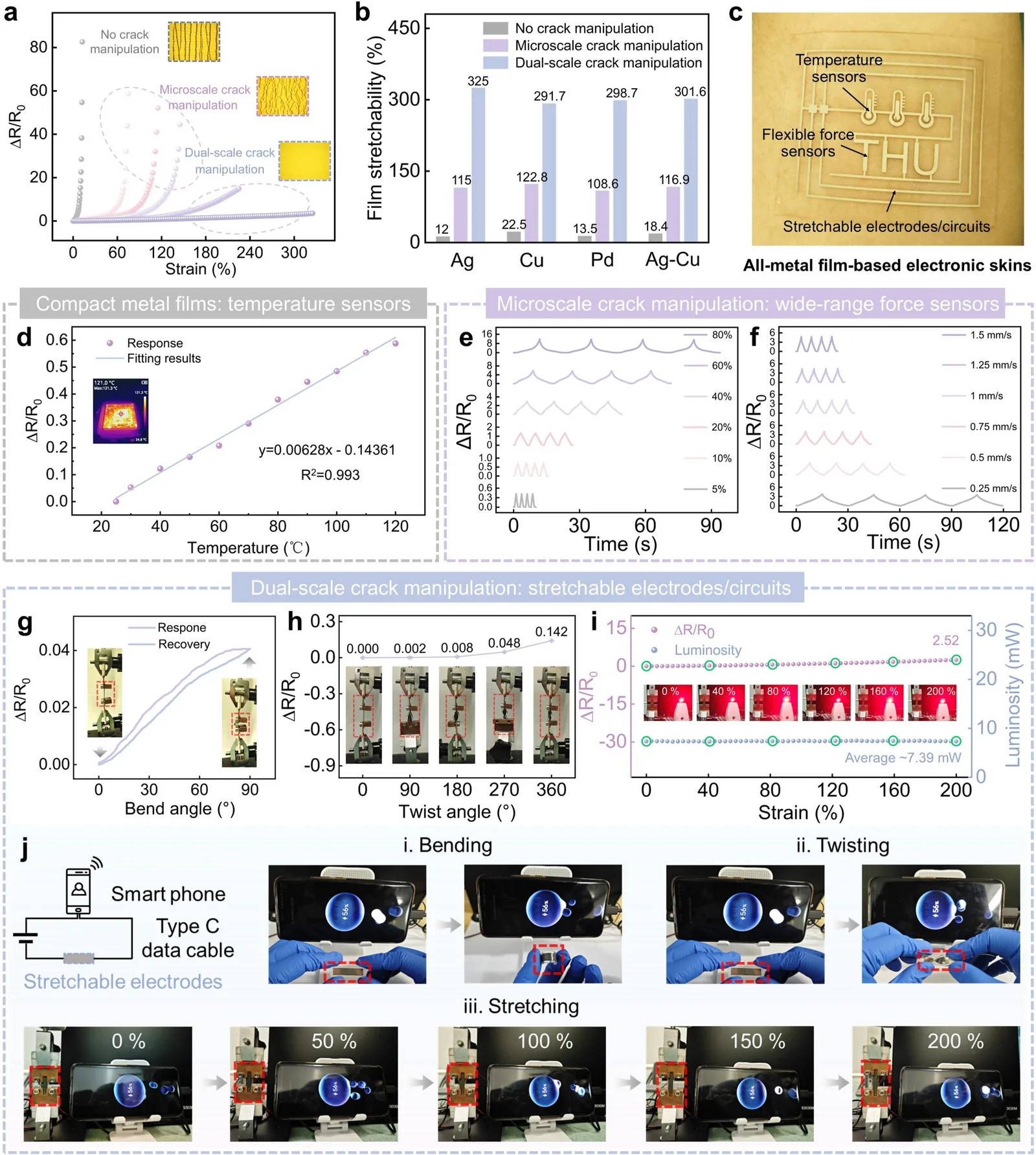
Electromechanical performance of the proposed dual-scale crack-manipulated metal films and their applications as versatile flexible components. **a** Relative resistance changes (Δ*R/R*_0_) *vs* strain for metal films under different crack manipulation strategies. Insets: crack patterns formed under no crack manipulation, microscale crack manipulation and dual-scale crack manipulation, respectively. **b** Broad applicability of the dual-scale crack manipulation strategy across different metal films. **c** All-metal-film-based electronic skin integrating temperature sensors, flexible force sensors, and stretchable electrodes/circuits via the crack-manipulated electromechanical performance regulation. **d** Metal film-based temperature sensors for temperature sensing from 25 to 120 °C. **e, f** Performance of metal film-based force sensors under various applied strain (loading rate: 0.5 mm s^−1^) and loading rates (applied strain: 50%), respectively. **g, h** Relative resistance changes (Δ*R/R*_0_) of metal film-based stretchable electrodes/circuits at different bending and twisting angles, respectively. Insets: experimental setups. **i** Relative resistance changes (Δ*R/R*_0_) and LED luminous intensity versus strain for a stretchable electrode in a closed-loop circuit. **j** Metal film-based flexible electrodes used as Type-C data lines for smartphone charging under stretching, bending, and twisting

The above findings demonstrate the evolution of crack patterns in strained metal films based on the dual-scale crack manipulation strategy. This substantial variation in cracking behavior enables a precise regulation of electromechanical performance. As shown in Fig. 3a, metal films deposited on smooth substrates at low deposition pressure (0.5 Pa) exhibit through-film cracking and experience electrical failure at low strain (12%). In contrast, metal films on microbump-roughened substrates show improved stretchability (~ 75%) via winding crack patterns. Further optimization of morphological features—such as increased crack density and reduced crack length/width—improves stretchability to ~ 145%, indicating suitability for flexible force sensors in detecting large deformations. On this basis, pressure-regulated nanopore implantation induces tiny-networked crack patterns that further improve stretchability: 225% at 300 Pa and 325% at 1000 Pa. Notably, metal films deposited at 1000 Pa exhibit an approximately linear strain-sensing curve, with a low relative resistance change (Δ*R/R*_*0*_ = 0.6) at 100% strain and a high *Q* value (~ 0.926) at maximum strain. Such extraordinary strain-insensitive behavior is rarely reported for deposited solid metal films and is comparable to that of inherently stretchable liquid metal materials (Fig. S20), highlighting their potential as stretchable electrodes/circuits [42, 50–55]. The stretchability and sensitivity gauge factor (GF, Note S4) of these metal films are further summarized in Fig. S21. Furthermore, this effective regulation of electromechanical performance by the dual-scale crack manipulation is applicable for common metal films, including Ag, Cu, Pd, and Ag–Cu alloy (Fig. 3b). To systematically analyze the relationship between crack microstructure and macroscopic electrical behavior, we developed a simplified electromechanical model that describes the strain-dependent resistance change in the dual-scale crack-manipulated metal films (Note S5).

These results highlight the ability to tailor the electromechanical performance of metal films for diverse roles: from highly strain-sensitive compact metal films to wide-range flexible force sensors, and even strain-insensitive flexible electrodes/circuits. Further, an all-metal-film electronic skin integrating these components was constructed as a proof of concept (Fig. 3c). The compact metal films feature a high gauge factor (GF = 56.3 at 0 ~ 6.0%, 378.0 at 6.0% ~ 10.8%, and 3391.3 at 10.8% ~ 12.0%; Fig. S23), a fast response speed (signal rising/falling time: 50/70 ms; Fig. S24), as well as a low detection limit (strain resolution: 0.1%; Fig. S25). Notably, their resistance is also sensitive to the environmental temperature [56, 57], enabling temperature sensing from 25 to 120 °C (Figs. 3d and S26, Note S6). The temperature-dependent resistance response of this sensor exhibits excellent linearity with an *R*^2^ value of 0.993. The slope of the fitted curve corresponds to the resistive temperature coefficient, yielding a sensitivity of 0.00628 °C^−1^. The measured response and recovery time of the sensor are 32 and 27 s, respectively (Fig. S27). As for the metal films deposited on surface-roughened substrates (i.e., only applying the microscale crack manipulation), they can function as wide-range strain sensors. As shown in Fig. 3e, the sensor exhibits repeatable and distinguishable resistance responses to 0 ~ 80% strain, thus offering broad prospects in motion monitoring and healthcare (more investigations in Sect. 4). The relative resistance change (Δ*R/R*_*0*_) remains almost constant within a loading rate range of 0.25 – 1.5 mm s^−1^ (Fig. 3f). Besides, the strain-resistance sensing curve can maintain a great repeatability for at least 1400 test cycles (Fig. S28). Post-cycling surface morphology analysis confirms that crack evolution generally stabilizes, which is consistent with the observed electrical stability (Fig. S29). The sensor also demonstrates satisfactory durability under representative wearable conditions, including exposure to high humidity (Fig. S30), sweat (Fig. S31), and temperature variations (Fig. S32). When microscale and nanoscale crack manipulation (i.e., surface-roughened substrates and high deposition pressure) are applied simultaneously, the metal film is capable for strain-insensitive and stretchable electrodes/circuits. The resistance remains relatively stable under various deformation conditions. The maximum resistance change is only 0.04 during the bending test at an angle of 0 ~ 90° (Fig. 3g). The proposed metal film also presents a low resistance change as the twist angle gradually increases from 0° to 360° (Fig. 3h). Moreover, the long-term cyclic stability is investigated by repeated cyclic testing under 150%. After 1000 stretching-release cycles, the initial resistance increases only slightly from 5.14–7.06 Ω, indicating a relatively stable electrical response with no abrupt degradation (Fig. S33). The tiny-networked crack pattern exhibits a high degree of reversibility during cyclic testing (Fig. S34). Irreversible crack propagation or catastrophic structural failure is rarely observed. This strain-insensitive flexible electrode/circuit for real-world application was systematically investigated (Fig. 3i, j). First, the metal films were connected with commercial light-emitting diode (LED) in series to construct a closed-loop circuit under a source voltage of 3 V (Fig. S35). Subsequently, the relative resistance change (Δ*R/R*_*0*_) of metal films and the LED luminous intensity were continuously recorded during stretching (0 ~ 200%). Throughout, the maximum resistance change was merely 2.52, while the LED luminous intensity remained constant at a value of 7.39 mW (Fig. 3i). Another interesting application scenario for flexible electrodes/circuits is as a component of Type C data lines during smartphone charging (Fig. 3j). The charging process maintained complete and continuous under diverse deformation situations such as stretching (0 ~ 200%), bending, and twisting. These results confirm that the proposed flexible electrodes/circuits with superior deformation tolerance and stable conductivity are promising for widespread flexible electronics. Finally, to facilitate a clear structure–property–function interpretation, Table S3 summarizes the causal mapping between structural design, crack pattern, electromechanical performance, and device-level functions.

### AI-Empowered Smart Sensing Application Based on the Proposed Crack-Manipulated Metal Films

**Fig. 4:**
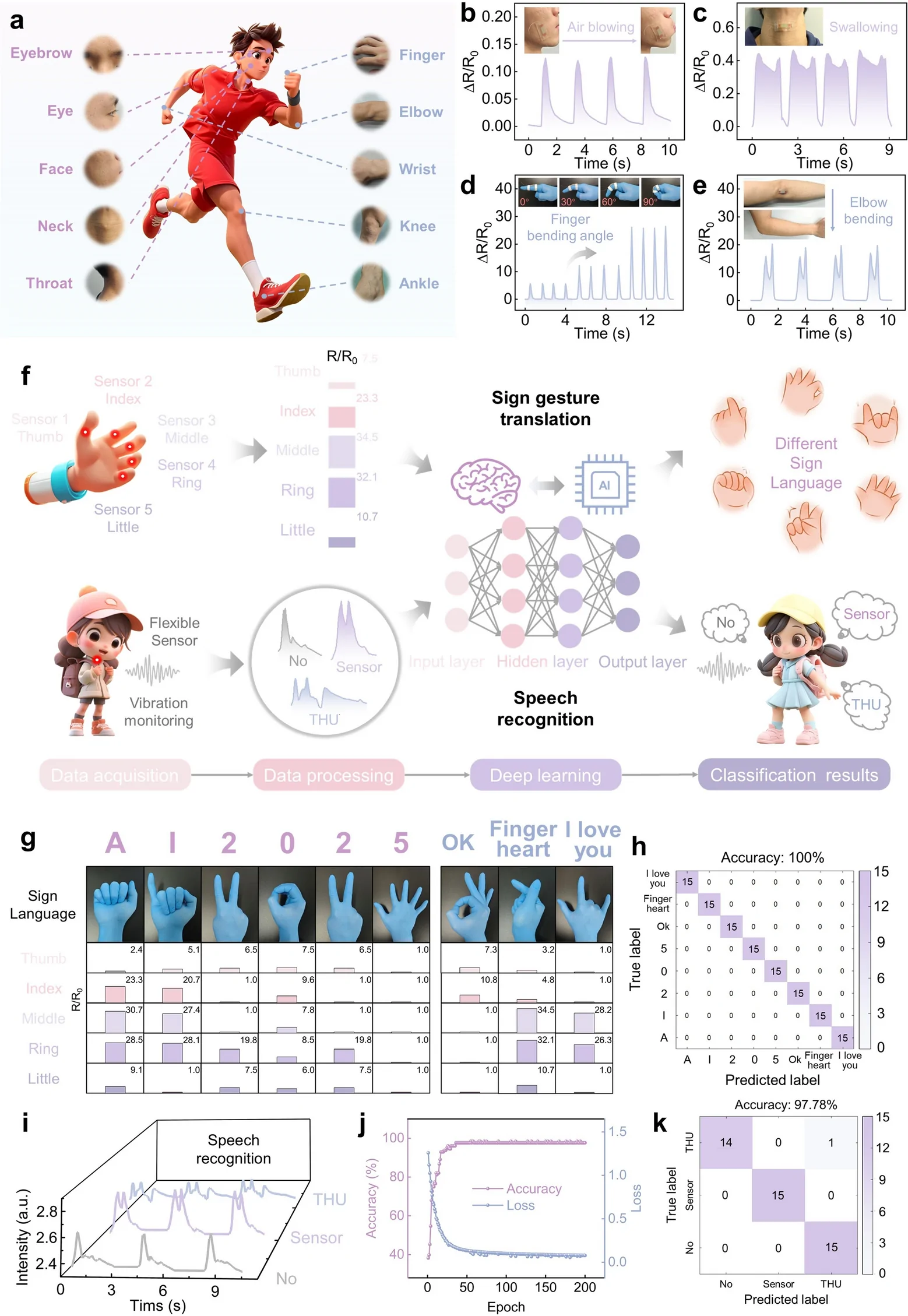
Application of the proposed metal film conductors for human motion monitoring and AI-empowered sensing. **a** Application of the sensors in human motion monitoring including facial activity monitoring and body motion sensing. **b, c** Facial activity monitoring: real-time resistance signals during air blowing and swallowing. **d, e** Body motion sensing: real-time resistance signals during finger bending at different angles and elbow bending. **f** Schematic of the CNN algorithm model for sign gesture translation and speech recognition. **g** Several typical gestures and their corresponding sensor signal intensity (*R/R*_0_) from five fingers. **h** Confusion matrix of the CNN model for the recognition results of various sign gestures. **i** Real-time signal response to various words or phrases. **j** Evolution of recognition accuracy and loss on the held-out test set over 200 training epochs for speech recognition. No early stopping or additional regularization was applied in this proof-of-concept study. **k** Confusion matrix of the CNN model for the recognition results of various words or phrases

The practicality of the proposed crack-manipulated metal films was investigated in detail. With substantially improved strain range, the metal film-based sensors were applied to human motion monitoring and healthcare, enabling facile detection of facial activities and body motions (Fig. 4a). First, the sensors were attached to facial regions, including the eyebrow, eye, cheek, neck, and throat. As volunteers performed motions (frowning, blinking, air blowing, swallowing, and neck movement), the corresponding resistance changes were precisely recorded (Figs. 4b, c and S36). The signals showed excellent reproducibility and were readily distinguishable. Similarly, the sensors attached to various joints (finger, elbow, wrist, knee, ankle) effectively monitored body motions. The bending-induced responses exhibited clear variations across different motions (Figs. 4d, e and S37). The sensors also demonstrated excellent mechanical robustness across different strain-frequency regimes (Figs. S38 and S39). These results highlight the potential of the proposed sensors in comprehensive motion detection.

Leveraging these valuable characteristics, we further explored sign language translation to assist individuals with disabilities. Five independent sensors were attached to each of the five fingers to capture the tensile strain signals generated by finger bending as the volunteer performed American Sign Language gestures (Fig. S40) [58]. To ensure consistency, these sensors were fabricated in the same batch under identical processing conditions, yielding statistically similar crack morphologies (Fig. S41) and electromechanical performance trends (Fig. S42), thereby minimizing mechanical and electromechanical variability. The normalized signal intensity (*R/R*_*0*_) from each sensor was recorded in real time, generating a distinct five-channel signal pattern for each gesture. This enabled the translation of gestures into character information, such as “AI 2025”, “Ok”, “Finger heart”, and “I love you” (Fig. 4g). Further, the deep learning algorithm was integrated to manage the collected sensing signals for accurate classification and recognition of various sign gestures. A convolutional neural network (CNN) model with data analysis and processing capabilities was constructed, which mainly consisted of an input layer, multiple convolutional layers, and an output layer [59]. The schematic of the CNN model for sign gesture translation is illustrated in Fig. 4f. Each executed gesture is represented by a data packet containing five independent sensor signals induced by finger bending. These sensor signals are processed by extracting the peak normalized resistance (*R/R*_*0*_) from each of the five channels, resulting in a five-dimensional feature vector that serves as the input to the CNN model. To ensure high classification and recognition accuracy, 480 valid datasets were collected, comprising eight gesture classes with 60 samples each. These raw datasets were randomly split into two parts, with 75% serving as the train set for the CNN model training and the remaining 25% serving as the test set to evaluate the accuracy of the trained model. The classification results on the held-out test set from multiple users are shown in Figs. 4h and S43, S44. The confusion matrices and test-set loss curves indicate that the model achieved over 95% recognition accuracy for various sign gestures after 200 training epochs under the current split and dataset size.

Beyond gesture translation, the sensors were applied to speech recognition, offering communication support for individuals with vocal impairments. The sensor was placed on the throat to dynamically capture high-frequency, low-amplitude periodic strain signals associated with muscle movement and sound vibrations. When the volunteer repeated different words or phrases, such as “No”, “Sensor”, “THU”, the signal waveforms exhibited distinct characteristics, which may reflect differences in syllable count (Fig. 4i) [60, 61]. This difference could arise from the physiological factors associated with muscle movement during speech and the acoustic factors of sound vibrations. The CNN model described above also enabled the accurate classification and recognition of various spoken words (Fig. 4f). For each speech sample, a 14‑dimensional feature vector is extracted to characterize the distribution statistics and intensity profile of the speech signal. This feature vector, comprising time domain features such as mean, standard deviation, skewness, kurtosis, maximum, minimum, peak-to-peak, root mean square, amplitude factor, form factor, crest factor, impulse factor, margin factor, and energy, serves as the input to the CNN model. We collected 180 data samples in total (60 per class) across three word/phrase classes. The dataset was randomly split into a training set (75%) and a testing set (25%). Over 200 training epochs, the classification accuracy improved steadily, ultimately achieving a test accuracy of over 95% on the held-out test set from multiple users for various words or phrases under the current data split and dataset size (Figs. 4j, k and S45). Although the current results for sign gesture translation and speech recognition rely on a proof-of-concept dataset with a limited number of samples, the achieved accuracy remains encouraging. By integrating crack-based flexible sensors with deep learning, this AI-enhanced gesture and speech recognition system shows promise for improving communication aids for deaf and mute individuals, promoting accessible technology and intelligent medical solutions.

## Conclusion

In summary, our work fundamentally reinterprets the role of cracking in metal films, transforming it from an unfavorable failure event into a powerful tool for on-demand tailoring of film properties. Based on a bioinspired hierarchical architecture, we achieved dual-scale crack manipulation in metal films by exploiting the synergistic interplay between nanoscale pore implantation and microscale substrate roughening. This approach enables a progressive transition in crack morphology, evolving from through-film to winding and finally to networked microcracks. As a result, a remarkable 25-fold regulation of stretchability (from 12% to 325%) was achieved, thereby converting traditionally brittle metal films into stretchable and tunable conductors that can function as either wide-range strain sensors or strain-insensitive electrodes/circuits. Demonstrating excellent universality across common metal systems (Ag, Cu, Pd, and Ag–Cu alloy), the practicality of this strategy was validated through a stretchable metal film-based, AI-empowered electronic skin. This integrated system exhibits robust performance in complex scenarios such as human motion monitoring, sign language translation, and speech recognition. Overall, this bioinspired multi-scale regulation paradigm for developing stretchable metal conductors not only opens a new avenue by leveraging size effects in crack engineering, but also accelerates the commercialization of flexible electronics.

## Supplementary Information

Below is the link to the electronic supplementary material.

Supplementary file1 (DOCX 17465 kb)
