# Supplementary material for: Bioinspired Dual-Scale Crack Manipulation Enabling 325%-Stretchable Metal Film Conductors for AI-Empowered Electronic Skins
Source: Nanomicro Lett. 2026 Apr 7;18:318. doi: 10.1007/s40820-026-02152-5 (PMC13057072; doi:10.1007/s40820-026-02152-5)
Supplement: Supplementary file 1 — Supplementary file1 (DOCX 17465 kb) [file 40820_2026_2152_MOESM1_ESM.docx]

Supporting Information for

**Bioinspired Dual-Scale Crack Manipulation Enabling 325%-Stretchable Metal Film Conductors for AI-Empowered Electronic Skins**

Tianming Sun^1, 2^ ‡, Bin Feng^3^ ‡, Guisheng Zou^1, 2,^ *, Jinpeng Huo^1, 2^, Bo Bi^1, 2^, Jin Peng^1, 2^, Zehua Li^1, 2^, Gongbo Bian^4^, Bingang Xu^3^, Lei Liu^1, 2,^ *

^1^ State Key Laboratory of Clean and Efficient Turbomachinery Power Equipment, Department of Mechanical Engineering, Tsinghua University, Beijing, 100084, China.

^2^ Key Laboratory for Advanced Materials Processing Technology, Ministry of Education, Beijing 100084, China.

^3^ Nanotechnology Center, School of Fashion and Textiles, The Hong Kong Polytechnic University, Hung Hom, Kowloon 999077, Hong Kong, China.

^4^ Ningbo Institute of Materials Technology & Engineering, Chinese Academy of Sciences, 315201, China.

‡ Tianming Sun and Bin Feng contributed equally to this work.

*Corresponding authors. E-mail address: [liulei@tsinghua.edu.cn](mailto:liulei@tsinghua.edu.cn) (Lei Liu); [zougsh@tsinghua.edu.cn](mailto:zougsh@tsinghua.edu.cn) (Guisheng Zou)

Note S1 **The elucidation of deposition pressure-regulated nanopore implantation in metal films**

In the physical vapor deposition process, deposition pressure is a key parameter that strongly affects the microstructure of the resulting thin films. As shown in Figure S8, with higher deposition pressure, nanopores emerge within the deposited metal film, accompanied with the increased the film porosity. The film is transformed from a compact to a loose structure. The underlying mechanism of this pressure-induced microstructure variations may involve a series of complex physical processes, including collisions, scattering, excitation and chemical reactions between high-energy particles generated by laser ablation and gas atoms during the deposition process [S1]. Several potential mechanisms have been proposed to explain the increased nanopores at higher deposition pressures. First, under high-pressure environments, the ejected atoms from the metal target undergo high-frequency collisions with gas atoms and possess shorter mean free paths, resulting in more random atomic scattering [S1, S2]. This enhanced atomic scattering induces disordered atomic stacking and facilitates the formation of massive nanopores. Second, high-frequency collisions substantially reduce the kinetic energy of the ejected atoms [S3]. As a result, these ejected atoms lack sufficient energy for migration and diffusion after adsorbing onto the substrate, which affects particle arrangement and increases nanopore density. Consequently, the overall structure of the deposited metal film becomes relatively loose.

Importantly, the above finding indicates that the formation of nanopore-implanted metal films is generally independent of the specific manufacturing technique. Therefore, although the nanoscale pore implantation strategy is demonstrated using pulsed laser deposition in this work, this strategy is promising to be extended to other methods, such as controlled atmosphere sputtering (via tuning working gas pressure, gas composition, and sputtering power), templated deposition that introduce sacrificial layer, or chemical approaches.

In our design, the three deposition pressures (0.5 Pa, 300 Pa, and 1000 Pa) were selected as key process parameters, covering three representative regimes of film growth/densification.

⚫ At 0.5 Pa, the long mean free path and high kinetic energy of the deposited particles enable sufficient surface migration, resulting in a compact film that tends to form long, straight through‑film cracks under strain.

⚫ At 300 Pa, the increased collision probability dissipates particle kinetic energy, promotes clustering, and restricts surface migration, yielding a film with moderate porosity that tends to form networked cracks under strain.

⚫ At 1000 Pa, the pronounced collisions and cluster‑based deposition produce a highly loose and porous film. This film tends to develop a fine network of microcracks, thereby effectively inhibiting catastrophic through‑film crack propagation.

Note S2 **Finite element simulations**

Finite element simulations (ABAQUS) were performed to resolve the stress and deformation fields of microbump-roughened PDMS substrates and Ag films. Two-dimensional finite element models were constructed for the Ag film and the PDMS substrate based on the actual specimen, respectively. The geometries were discretized using triangular elements with a refined mesh size to ensure convergence, resulting in 20389 elements for the Ag film and 11174 elements for the PDMS substrate. To reduce computational cost while preserving the essential physics of stress transfer, both materials were modeled as isotropic and homogeneous continua. Material parameters (Young’s modulus and Poisson’s ratio) were taken from established references and the standard ABAQUS material database [S12,32]. In the initial load-transfer analysis, linear elastic behavior was assumed for the material. The boundary conditions were defined to closely mimic the experimental tensile configuration. For the Ag film, the left boundary was fully fixed, while a prescribed tensile strain was applied to the right boundary. To approximate the plane-strain condition commonly assumed for thin films constrained by a compliant substrate, the vertical displacements of the top and bottom edges were restricted. Pre-existing defects were also introduced into the film geometry to analyze stress redistribution. Similarly, for the microbump-roughened PDMS substrate, one end was fixed and the opposite end was subjected to a prescribed tensile strain. The bottom surface was constrained in the vertical direction to represent rigid support, while the top surface was allowed to deform freely, enabling the response of surface microstructures to be captured.

This simplified model qualitatively maps the stress gradients and identifies preferential directions for crack driving forces introduced by the structural design. By isolating key geometric and mechanical variables, it offers a qualitative, mechanism explanation of the experimental trends, rather than aiming for precise quantitative agreement.

Note S3 **The concept of four normalized parameters: crack density, cut-through proportion, vertical offset, and horizontal/vertical offset ratio**

Four normalized parameters (crack density, cut-through proportion, vertical offset, and horizontal/vertical offset ratio) were employed to evaluate the features of the crack pattern of each case in Figure 2a. The films deposited on smooth substrates at low pressure (0.5 Pa) were set as the base level.

Crack density was defined as the number of effective cracks within the entire optical field of view. Cut-through proportion was the ratio of cracks that traverse the entire image to the total number of cracks. The concepts of vertical offset and horizontal offset of the crack were illustrated schematically in Figure S13. Vertical offset was defined as the maximum displacement of a crack along the vertical axis during its propagation. Horizontal/vertical offset ratio refers to the ratio of the maximum horizontal displacement to the corresponding maximum vertical displacement of a crack during propagation.

Note S4 **Gauge factor (GF) in flexible electronics**

In flexible electronics, the gauge factor (GF) is a key parameter that quantifies the capability of strain sensors to convert mechanical strain into an electrical signal. It reflects the device sensitivity and is defined as the ratio of the relative resistance change to the applied strain: GF=(*ΔR/R_0_*)/*ε* [S4, S5]. Here, *ΔR* represents the resistance change of metal films under strain, *R_0_* is the initial resistance of metal films, and *ε* denotes the applied strain. However, the sensing performance curves of metal films typically exhibit nonlinear characteristics. Therefore, the overall characteristic curve is divided into several quasi-linear segments, and the segment with the maximum GF value is defined as the sensor sensitivity. Further, the overall GF is defined as *(ΔR_tot_/R_0_)/ε_tot_*, where *ε_tot_* is the whole working range, and *ΔR_tot_* is the total resistance change[S6]. The overall GF provides a sense of how sensitive the sensor is to strain across its entire work range, rather than only at a localized strain window.

Note S5 **Proposed electromechanical model to elucidate the relationship between crack microstructure and macroscopic electrical behavior**

As shown in Figure 2a, the dual-scale crack manipulation strategy was proposed to induces a progression of crack patterns, from through-film cracks to winding cracks and ultimately to tiny-networked cracks in the strained metal films. These distinct crack patterns inevitably influence the electromechanical performance of metal films.

To systematically analyze the relationship between crack microstructure and macroscopic electrical behavior, we developed a simplified electromechanical model that describes the strain-dependent resistance change in the dual-scale crack-manipulated metal films. The evolution of the crack pattern clearly reveals that the long, straight cracks gradually transition into shorter, curved cracks. These cracks become increasingly finer and more densely distributed, with a corresponding increase in crack line density (Figure S22). This evolution originates from the stress concentration induced by the introduced microscale microbumps and nanoscale pores, which act as preferential sites for crack initiation under strain.

To quantitatively link microstructure to macroscopic electrical response, we considered the following framework. First, in a cracked metal film, the total resistance is dominated by the resistance contribution from crack regions, as the resistance of the remaining intact metal regions is negligible. The resistance of an individual crack region is described using a widely adopted electron tunneling model [S7–S9]:

(S1)

$$R_{crack}=\frac{h^{2}d}{Ae^{2}\sqrt{2m\phi}}\exp\left( \frac{4\pi d}{h}\sqrt{2m\phi} \right)$$

Where *h* is Planck’s constant, *d* is the average crack width, *Φ* is the energy barrier height, *A* is the effective cross-sectional area of the tunneling zone, *m* and *e* are the electron mass and charge, respectively. Second, under uniaxial tensile loading, the applied strain is largely accommodated by the widening of these crack gaps due to stress concentration (Figure 2c, d). We define the crack line density 𝑁 as the number of cracks per unit film length. Assuming the average crack width $d$is proportional to the applied strain $\varepsilon$ and inversely proportional to the crack line density $N$:

$d=k\frac{\varepsilon}{N}$ (S2)

where $k$ is a proportionality coefficient and $\varepsilon$is the applied strain. Substituting Equation S2 into Equation S1 yields:

$R_{crack}=\frac{h^{2}d}{Ae^{2}\sqrt{2m\phi}}\exp\left( \frac{4\pi d}{h}\sqrt{2m\phi} \right)=k_{1}\frac{\varepsilon}{N}exp(k_{2}\frac{\varepsilon}{N})$ (S3)

where $k_{1}$and $k_{2}$are lumped coefficients incorporating material and geometric constants.

The total resistance of a cracked metal film is determined by the cumulative contribution of all crack regions. Accordingly, the strain-dependent total resistance can be expressed as:

${R_{total}=R}_{crack}*N*L =k_{1}\varepsilon Lexp(k_{2}\frac{\varepsilon}{N})$ (S4)

where $L$ is the film length. This expression indicates that the strain-dependent resistance change is primarily governed by the characteristic parameter $N$ (crack line density). Since stress concentration sites induce crack initiation, the crack line density $N$is positively correlated with the density of these sites$P_{s}$. In the microscale design, finite element analysis reveals significant stress concentration at the base of microbumps. Therefore, $P_{s}$ is positively correlated with microbump density, which is controlled by the grit size of the sandpaper templates. In the nanoscale design, implanted nanopores also introduce localized stress concentration sites, making $P_{s}$positively correlated with nanopore density, which is tuned by deposition pressure.

Combining these analyses, an increased crack line density $N$at a given strain effectively reduces the total film resistance, consistent with the experimental observations (Figure 3a). The crack line density $N$is positively correlated with the density of stress concentration sites $P_{s}$, which is jointly determined by the microscale surface morphology (regulated by sandpaper grit size) and the nanoscale pore density (regulated by deposition pressure). These two factors synergistically govern crack initiation density from the microscale and nanoscale perspectives, respectively. Thus, the proposed dual-scale crack manipulation strategy effectively controls crack line density through the synergistic effects of microscale substrate roughening and nanoscale pore implantation, enabling effective and predictable regulation of the electromechanical performance of metal films.

Note S6 **Working principle of metal film-based temperature sensors**

In our research, the electrical resistance of compact metal films is sensitive to environmental temperature, making them suitable candidates for temperature sensing. The temperature sensing capability in such metal films relies on the resistive temperature coefficient (TCR), a fundamental bulk material property where electrical resistance changes predictably with temperature [S10, S11]. This relationship can be expressed as:

$R\left( T \right)=R_{0}\left[ 1+\alpha(T-T_{0}) \right]$ (S5)

Where *R(T)* is the resistance at temperature *T*, *R_0_* is the resistance at a reference temperature *T_0_* and *α* is the temperature coefficient of resistance (°C^-1^), which is typically positive for metals. The increase in resistance with temperature stems from enhanced electron scattering due to intensified lattice vibrations.

**Supporting Figures**


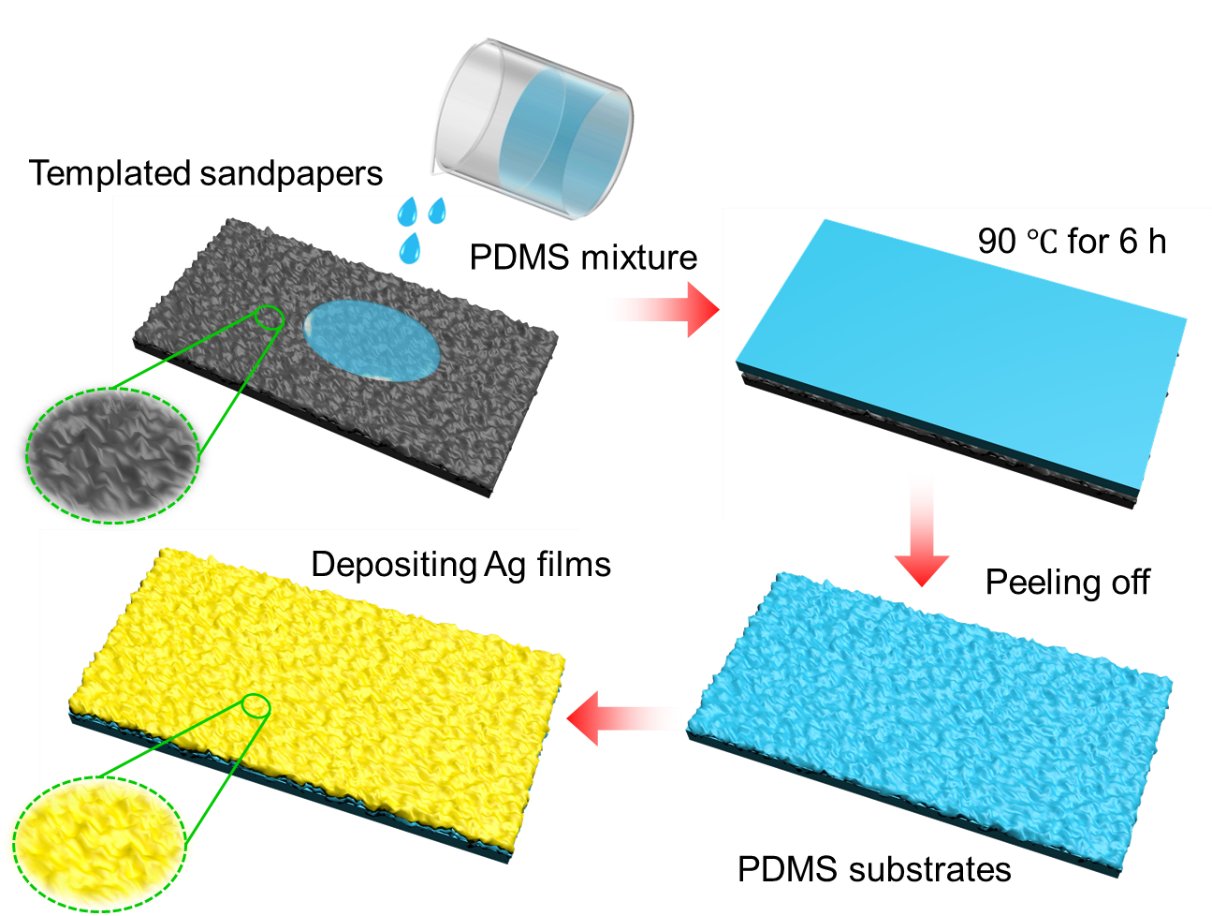


Fig. S1 The fabrication process of surface-roughened PDMS substrates


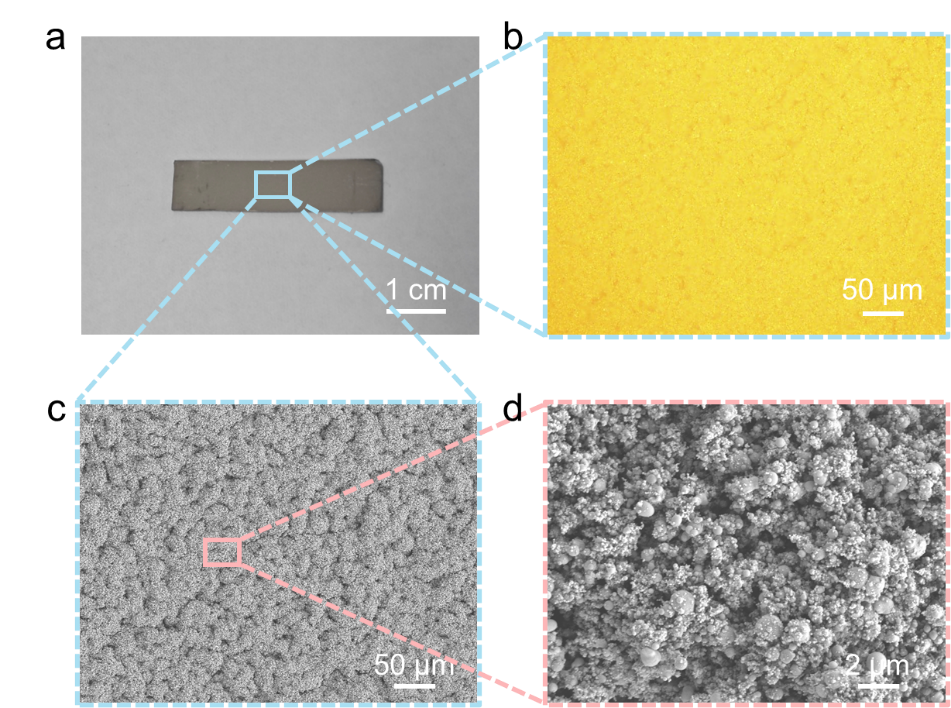


Fig. S2 The micromorphology of the dual-scale crack-manipulated metal films. (**a**) The photograph of nanopore-implanted metal films deposited on surface-roughened PDMS substrates, (**b**) The enlarged optical micrograph of the region in (a), (**c**) The SEM image of a representative metal film from (a), (**d**) The high-magnification SEM image of the region in (c)


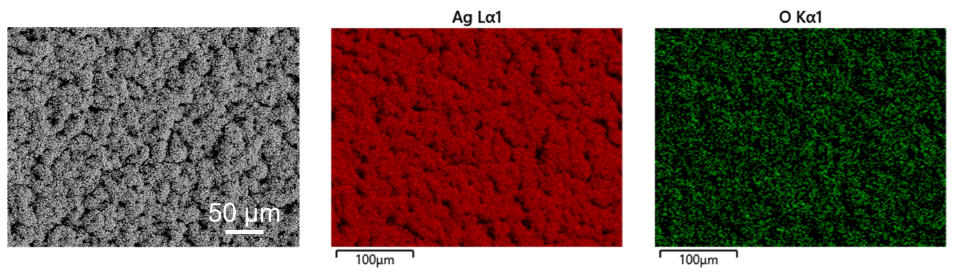


Fig. S3 The energy dispersive X-ray spectrometer (EDS) compositional analysis of as-deposited metal films


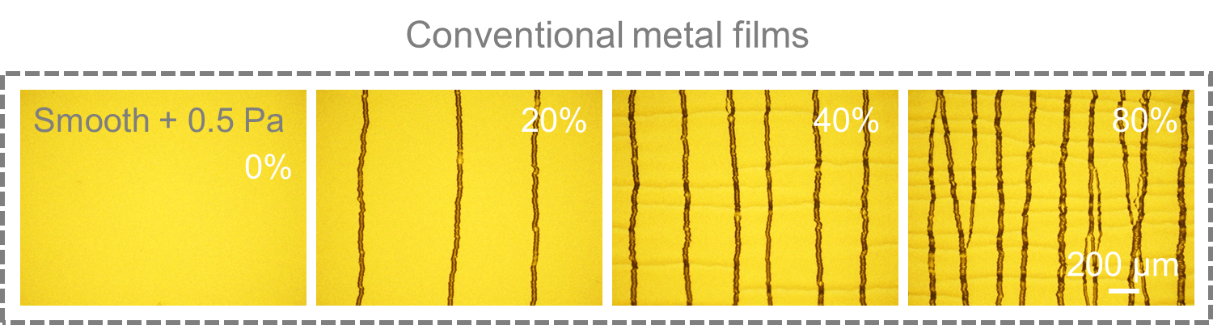


Fig. S4 The surface morphology evolution of conventional metal films deposited on smooth substrates at low pressure (0.5 Pa) under applied strain from 0% to 80%


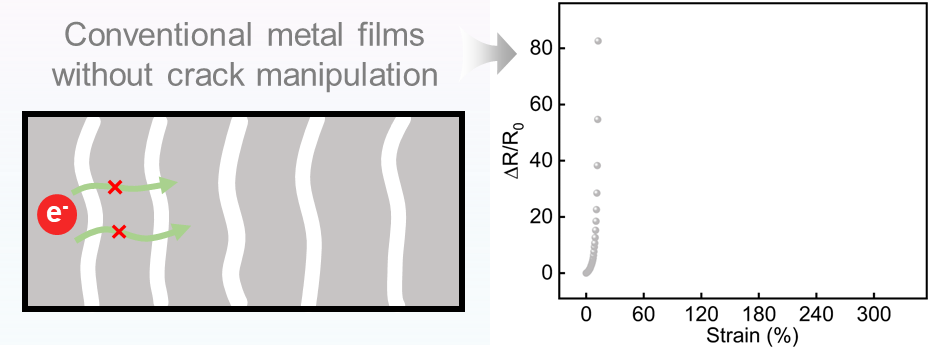


Fig. S5 Electrical transport model of metal films on microbump-roughened PDMS substrate under strain


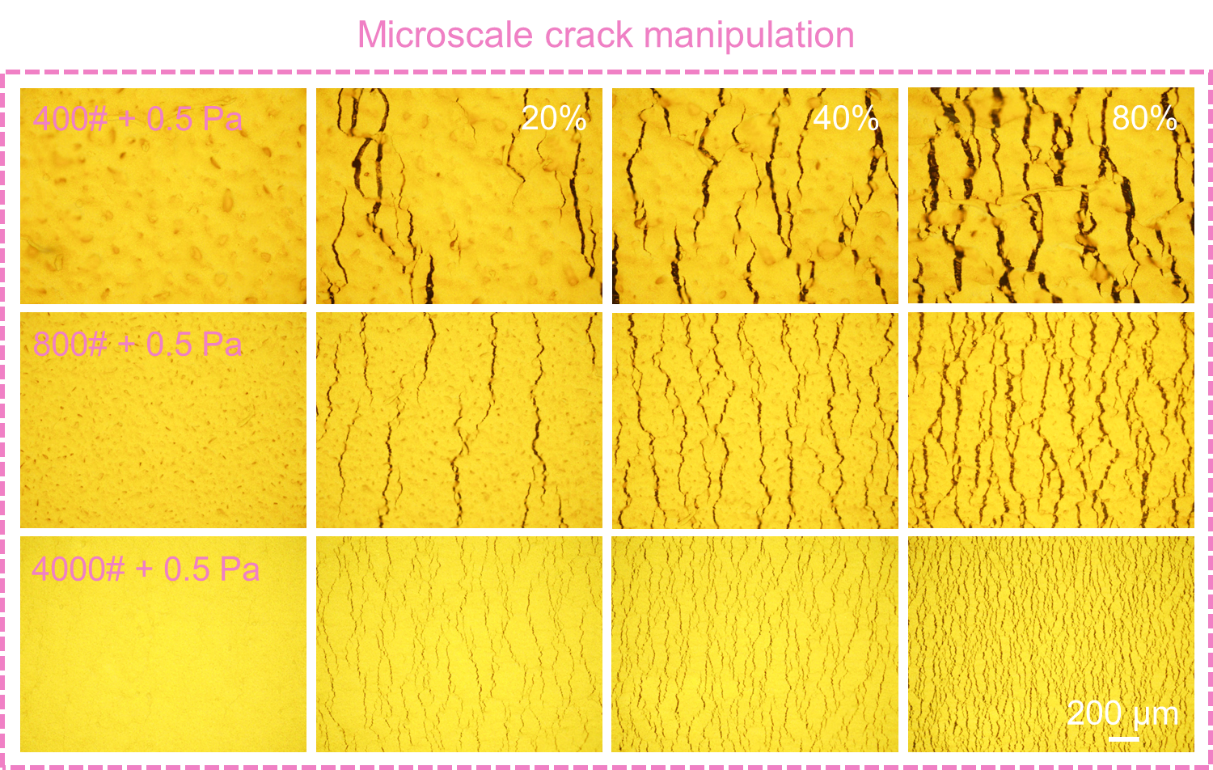


Fig. S6 The strain-dependent evolution of metal film morphology on microbump-roughened PDMS substrates with sandpaper grits of 400#, 800#, and 4000#. The top metal film remains a compact structure (deposition pressure: 0.5 Pa)


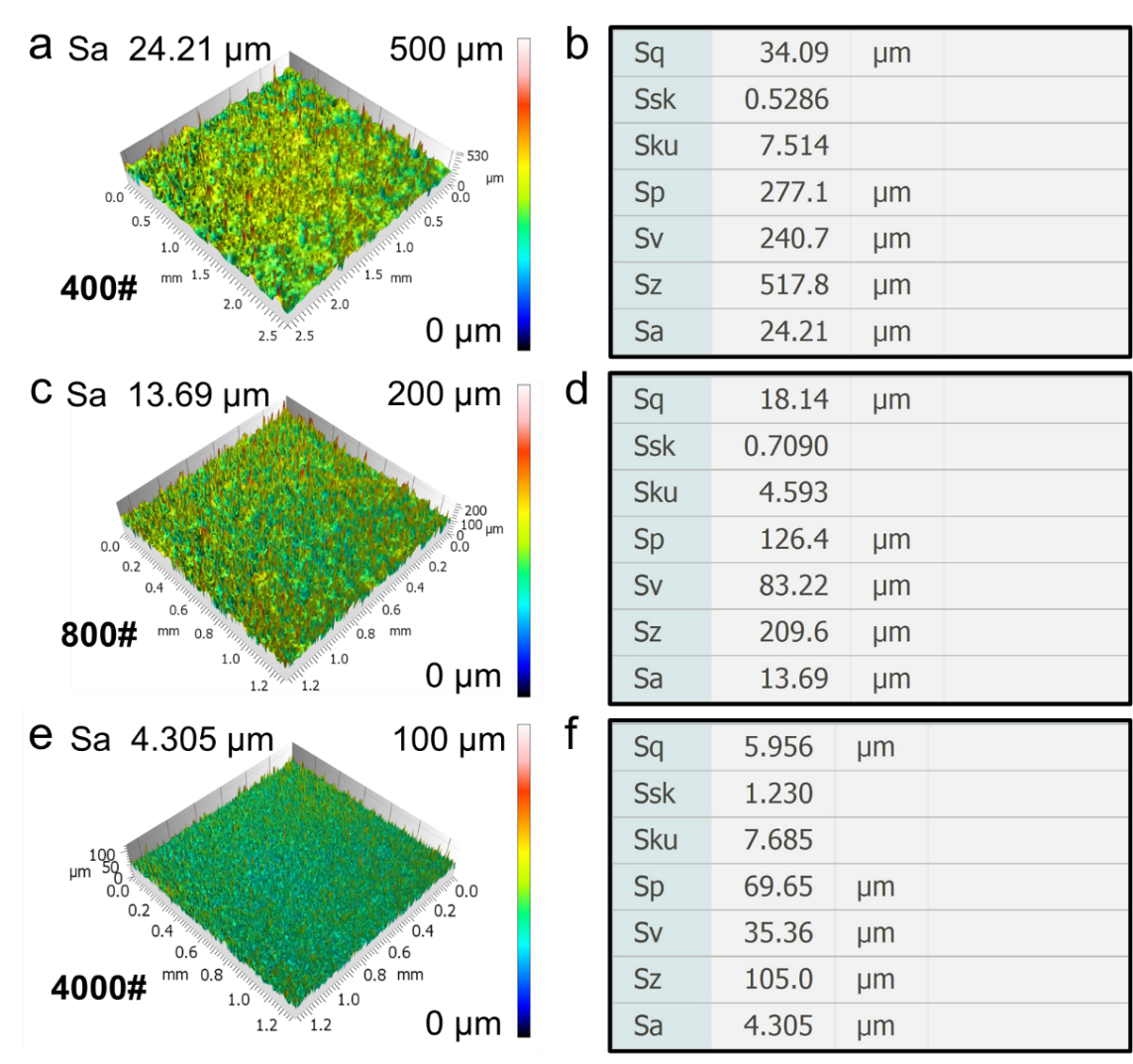


Fig. S7 The surface morphology of sandpaper with grits 400#, 800#, and 4000#


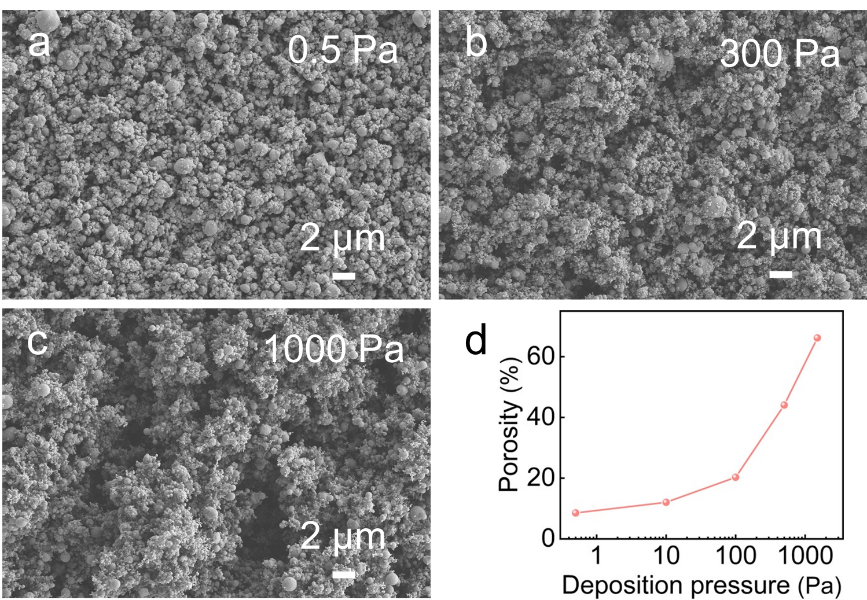


Fig. S8 The effect of deposition pressure on the film microstructure


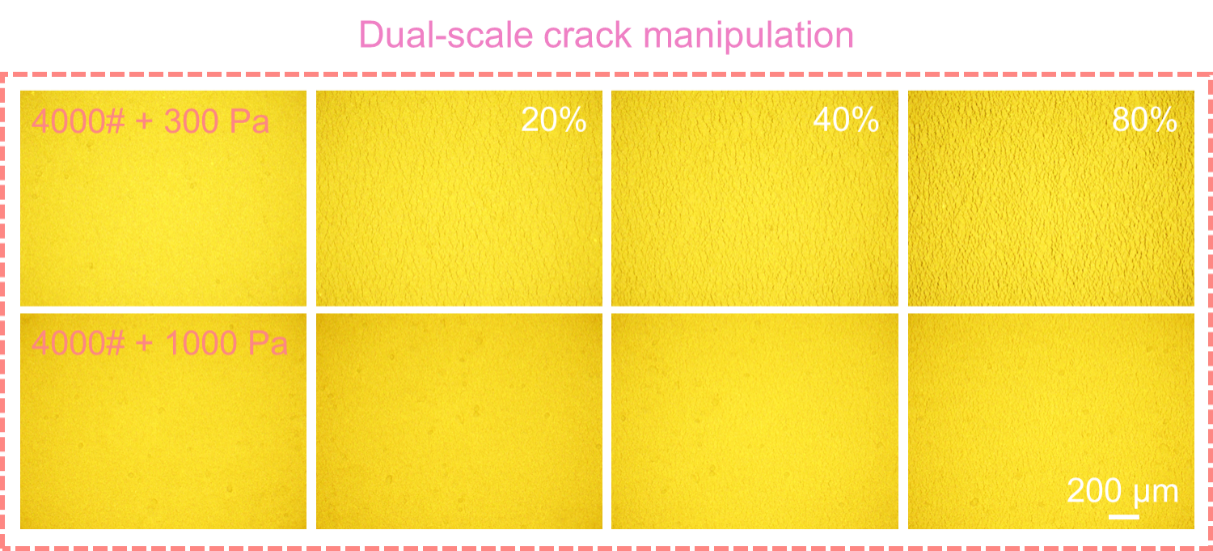


Fig. S9 The strain-dependent morphological evolution of nanopore-implanted metal films on a microbump-roughened PDMS substrate (deposition pressure: 300 Pa and 1000 Pa; sandpaper grits: 4000#)


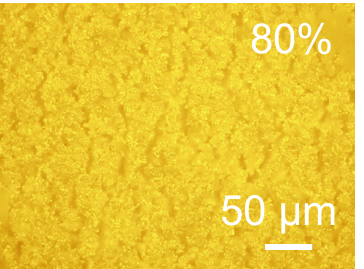


Fig. S10 High-magnification optical images (5-fold higher magnification than Figure 2a-iii) showing crack morphology in nanopore-implanted metal films on a microbump-roughened PDMS substrate at 80% applied strain (deposition pressure: 1000 Pa; sandpaper grits: 4000#)


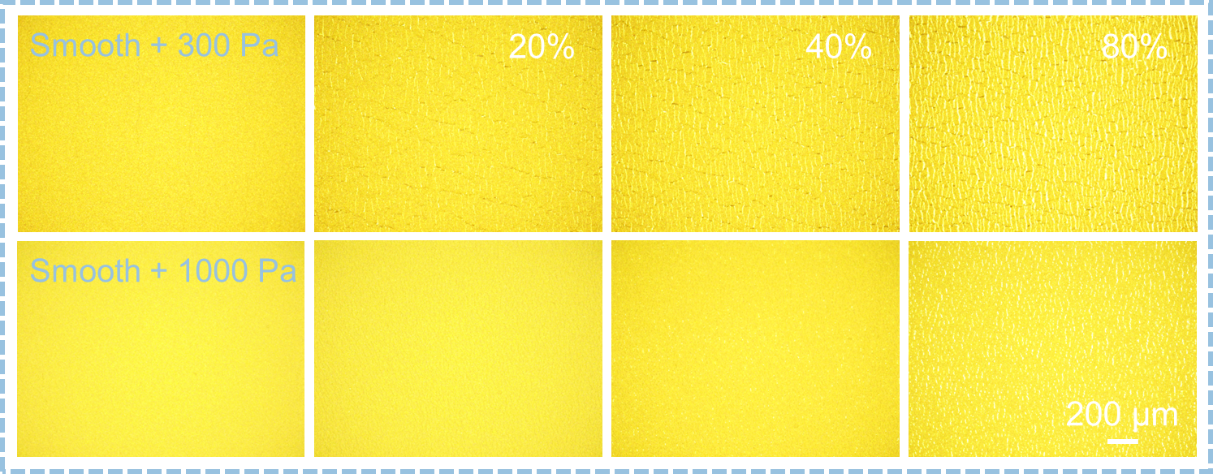


Fig. S11 The strain-dependent morphological evolution of nanopore-implanted metal films without microscale roughening (deposition pressure: 300 Pa and 1000 Pa)


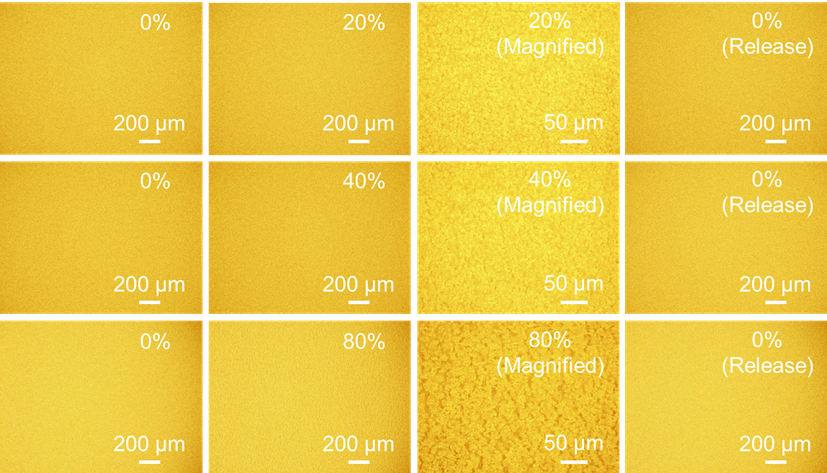


Fig. S12 The surface morphology of the dual-scale crack-manipulated metal films at different strain levels (20%, 40%, and 80%), both before stretching and after strain release. The third column shows the 5-folds higher magnification images (50x microscope) of samples


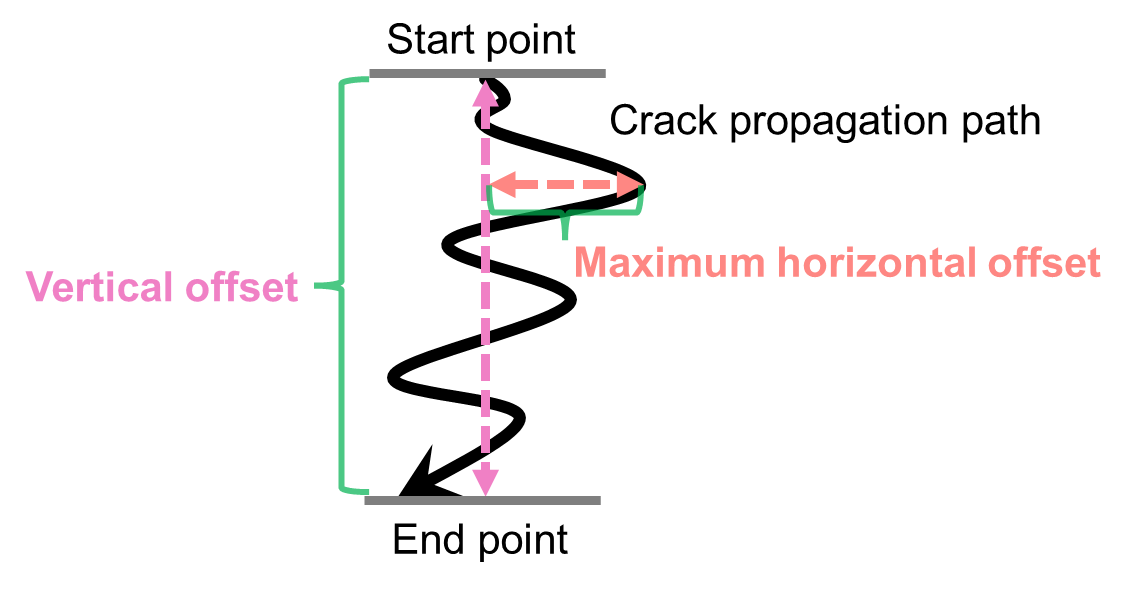


Fig. S13 The schematic illustration of the concepts of vertical offset and maximum horizontal offset of the crack
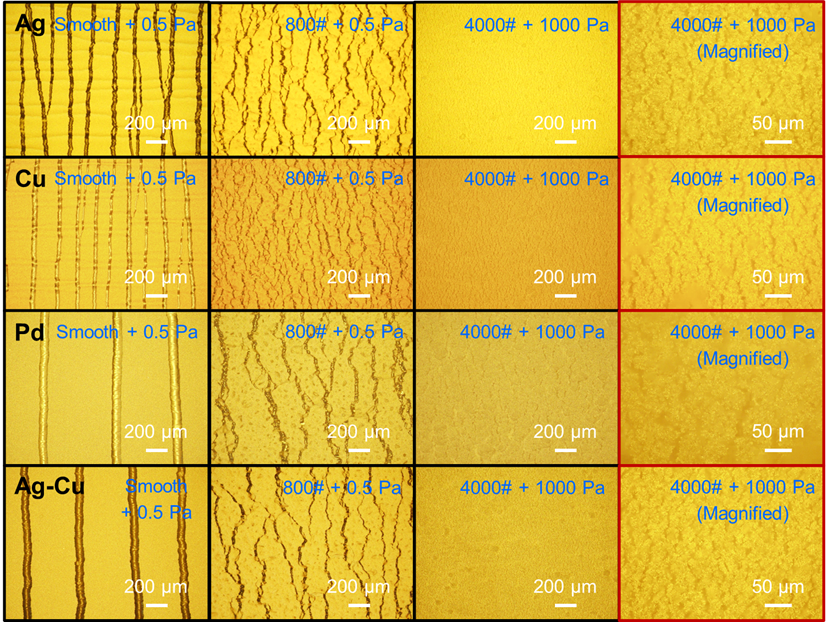


Fig. S14 The surface morphology of common metal films (Ag, Cu, Pd, and Ag-Cu alloy) under 80% strain based on the dual-scale crack manipulation strategy. The last column shows the 5-folds higher magnification images (50x microscope) of samples


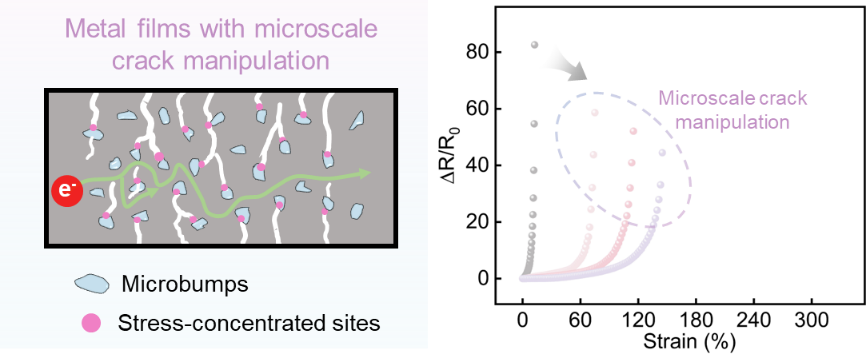


Fig. S15 Electrical transport model of metal films on microbump-roughened PDMS substrate under strain


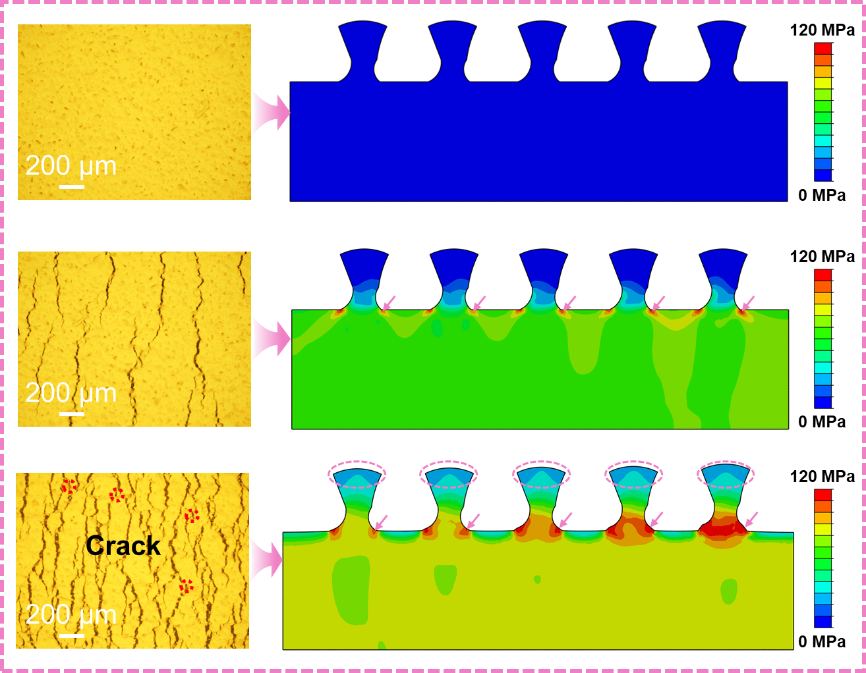


Fig. S16 The Von Mises stress distribution in compact metal films deposited on microbump-roughened substrates under stretching


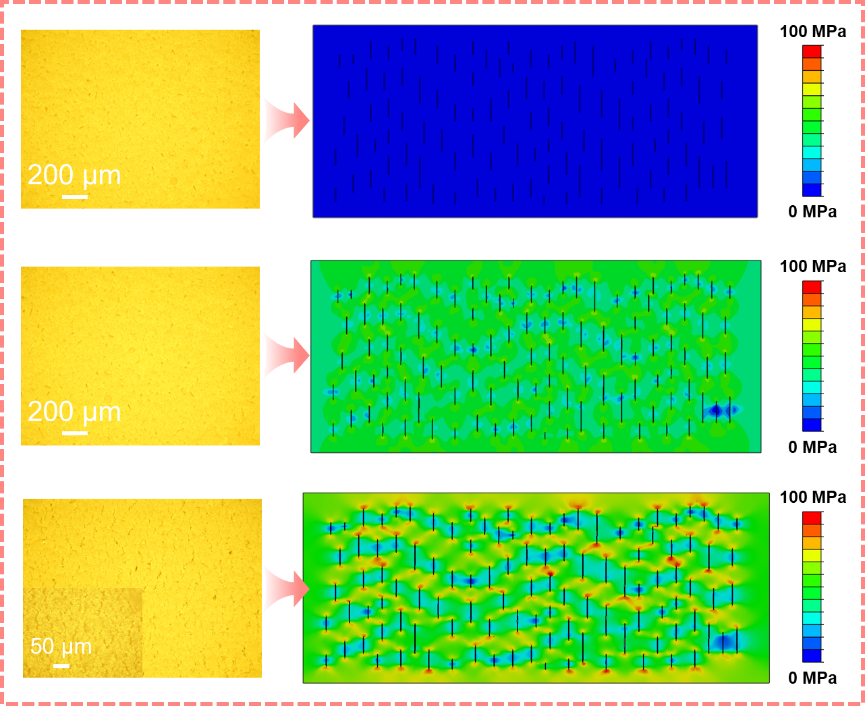


Fig. S17 The Von Mises stress distribution in nanopore-implanted metal films under stretching


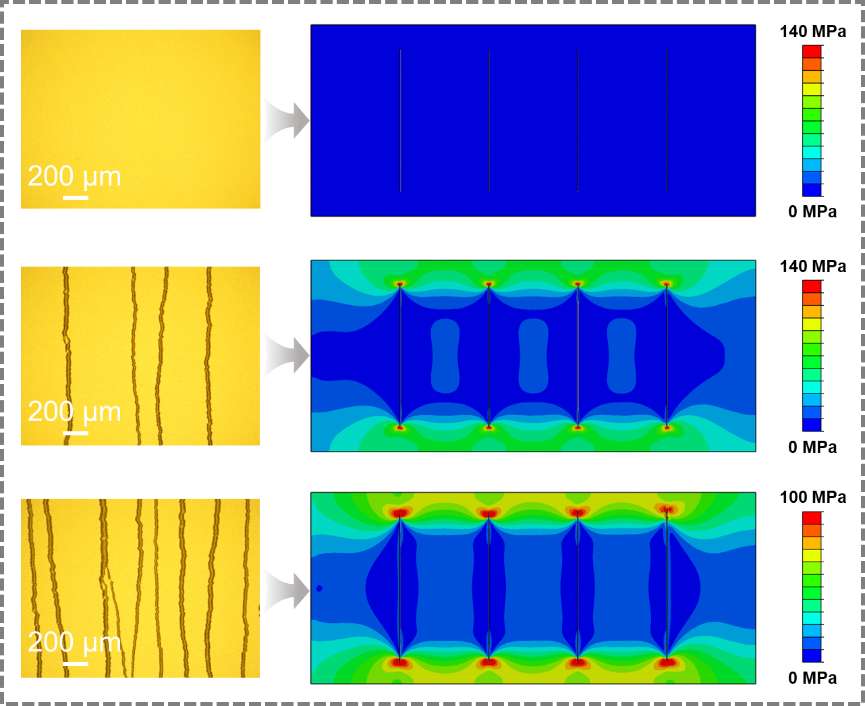


Fig. S18 The Von Mises stress distribution in compact metal films deposited on smooth substrates with few intrinsic defects under stretching


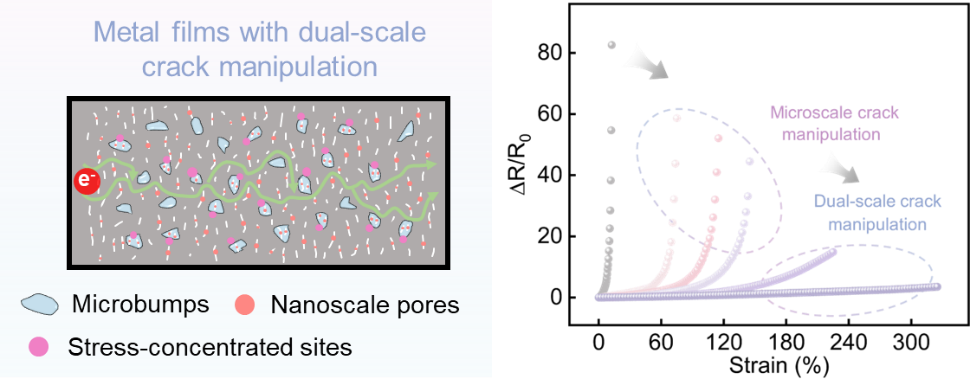


Fig. S19 Electrical transport model of nanopore-implanted metal films on microbump-roughened PDMS substrate under strain


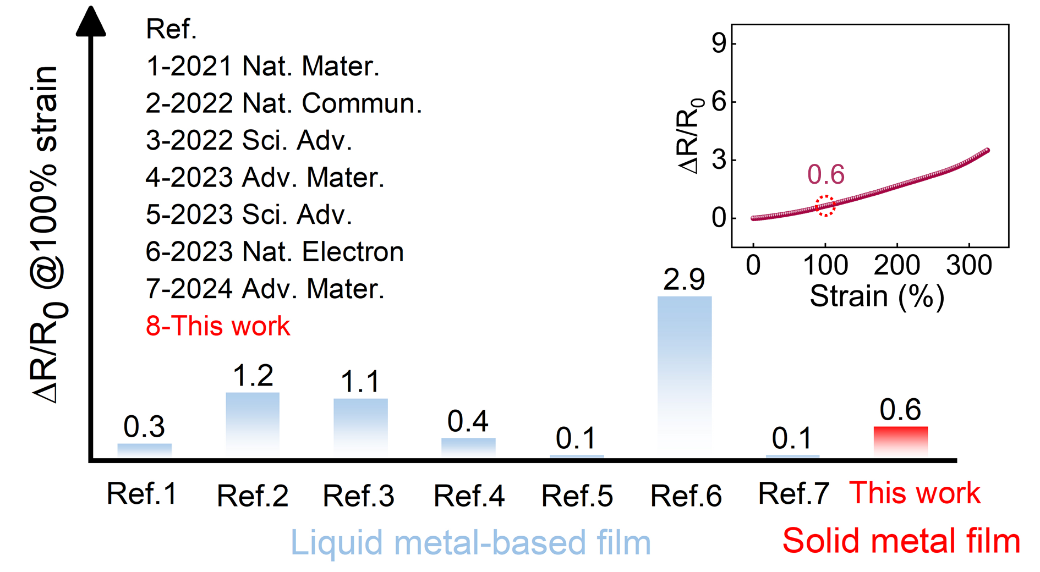


Fig. S20. The comparison of the relative resistance changes (*ΔR/R_0_*) at 100% strain for dual-scale crack-manipulated metal films and previously reported liquid metal-based films [S6, S12–S17]. The inset shows the relative resistance changes of the proposed metal films *vs* applied strain.


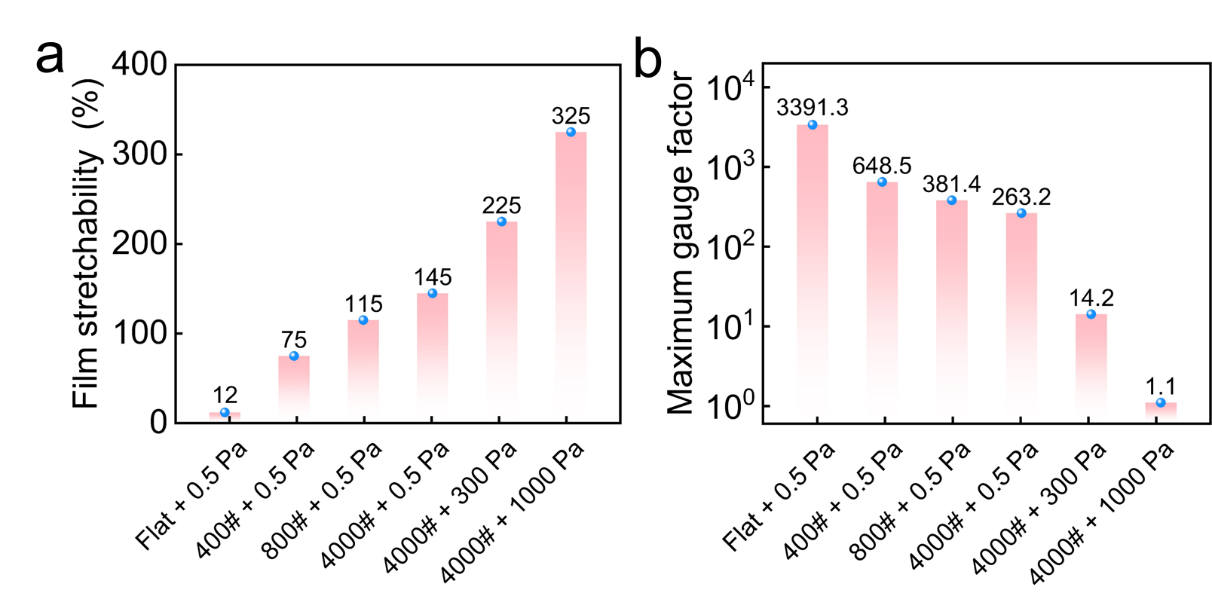


Fig. S21 The film stretchability and maximum gauge factor (GF) for metal films under different crack manipulation strategies shown in Figure 3a


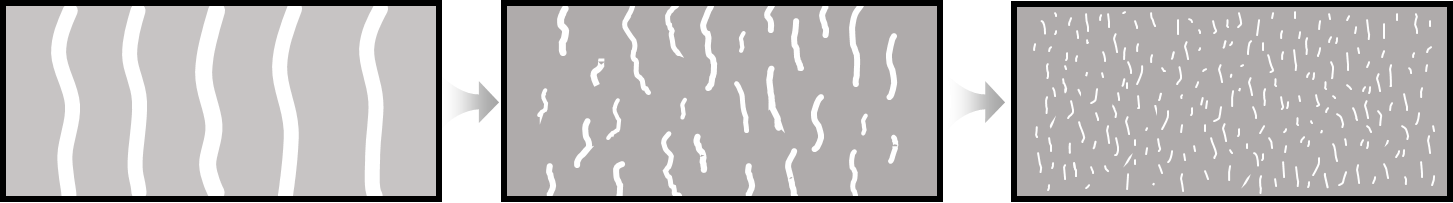


Fig. S22 Schematic of the crack pattern evolution indicates increased crack line density


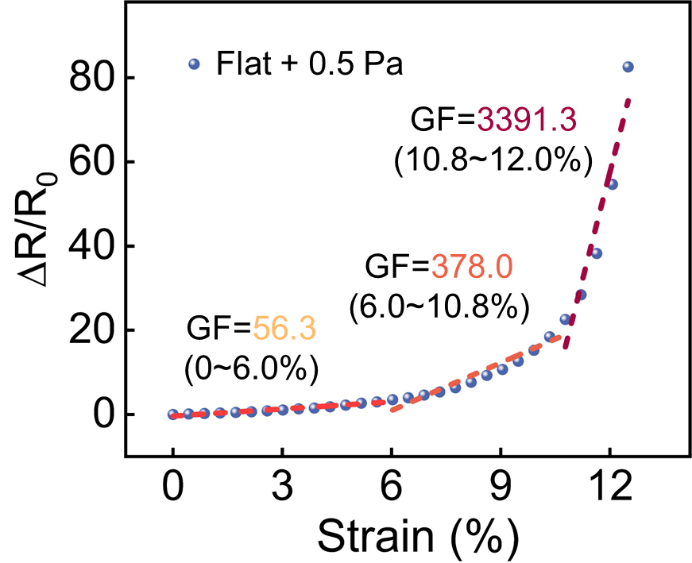


Fig. S23 The staged GF of highly strain-sensitive metal films


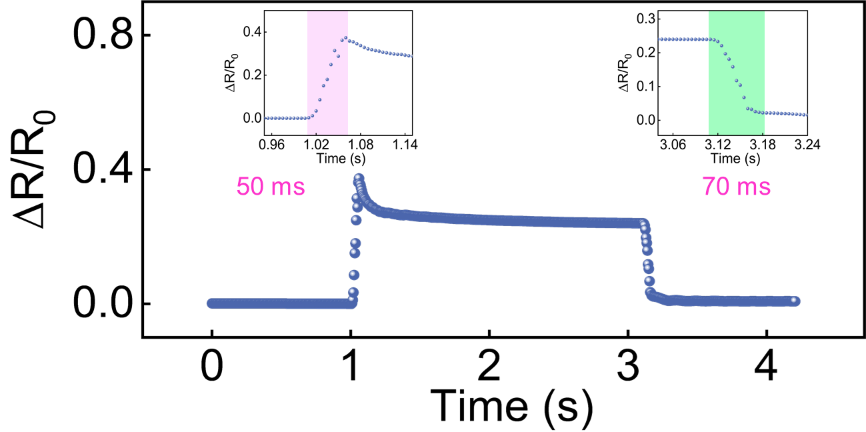


Fig. S24 The signal rising and falling time of highly strain-sensitive metal films (loading strain: 1%, loading speed: 10 mm s^-1^)


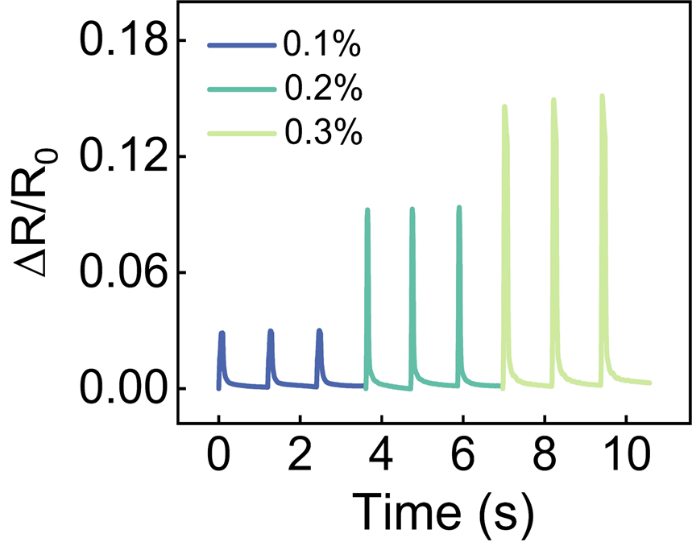


Fig. S25 The sensing capability to distinguish 0.1% strain variations for highly strain-sensitive metal films


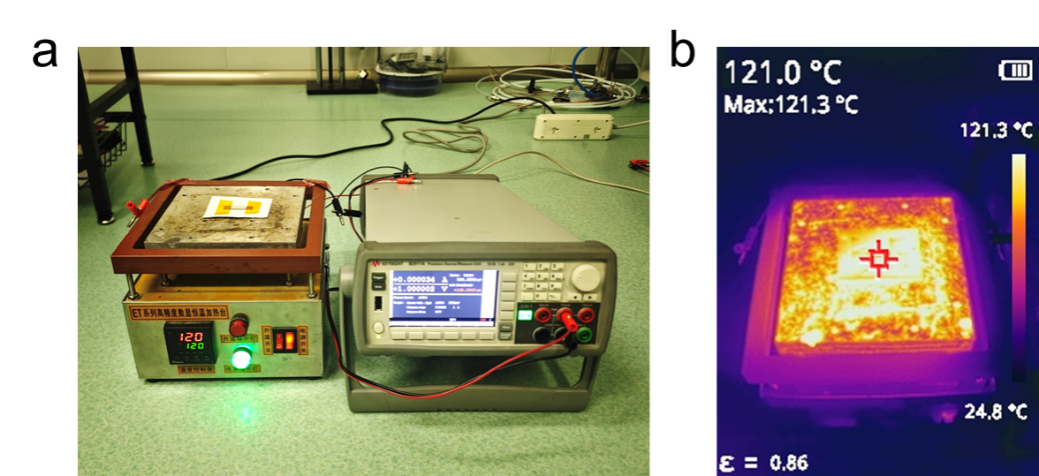


Fig. S26 The experimental setup for temperature sensor testing and the corresponding infrared temperature measurement results


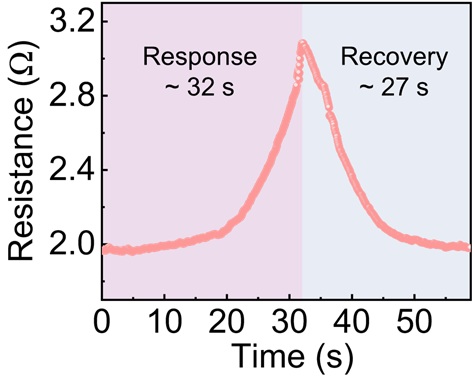


Fig. S27 The response and recovery time of the proposed temperature sensors between 25 °C and 120 °C


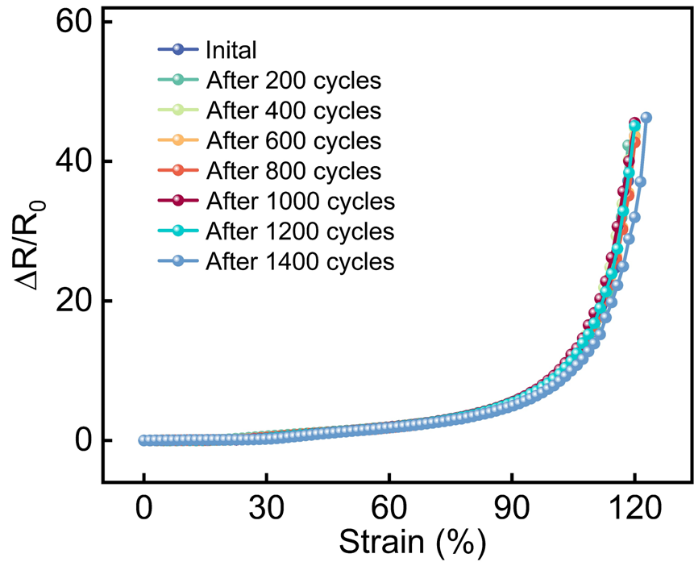


Fig. S28 The electromechanical performance of the wide-range flexible strain sensor after repeated cyclic loading (loading strain: 50%, loading speed: 0.5 mm s^-1^)


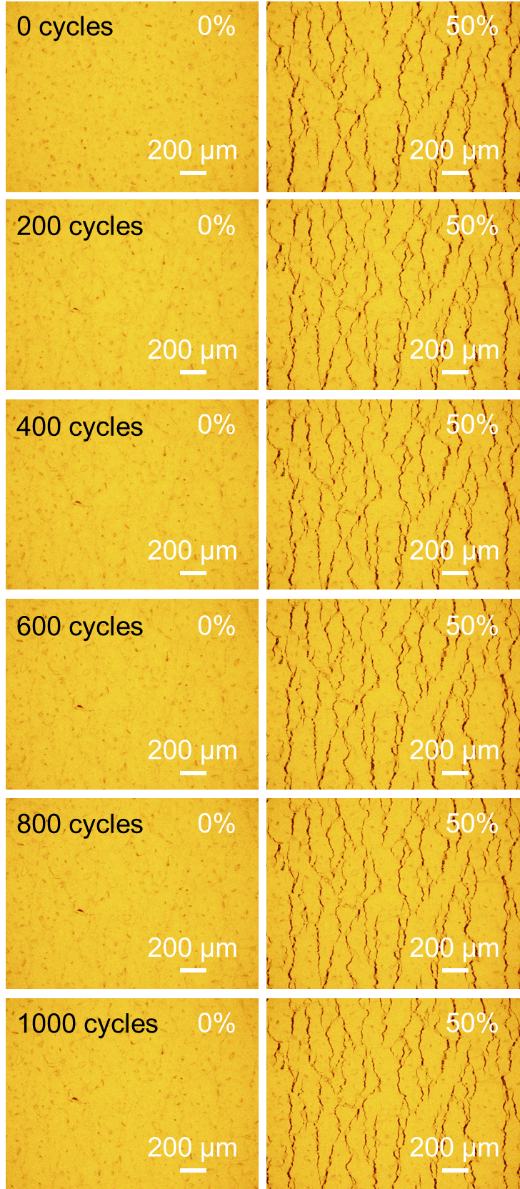


Fig. S29 The surface morphology of the metal film located on microbump-roughened substrates during cyclic testing


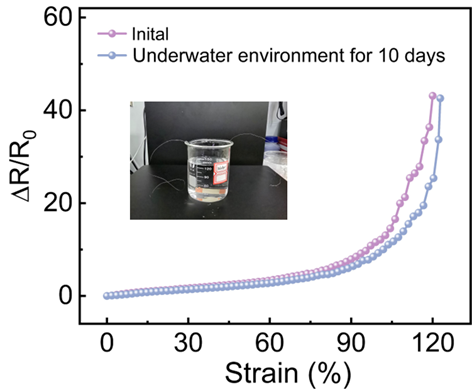


Fig. S30 The electromechanical performance of the metal film-based sensor before and after immersion in an underwater environment for 10 days. The inset shows the environment in which the sensor was placed


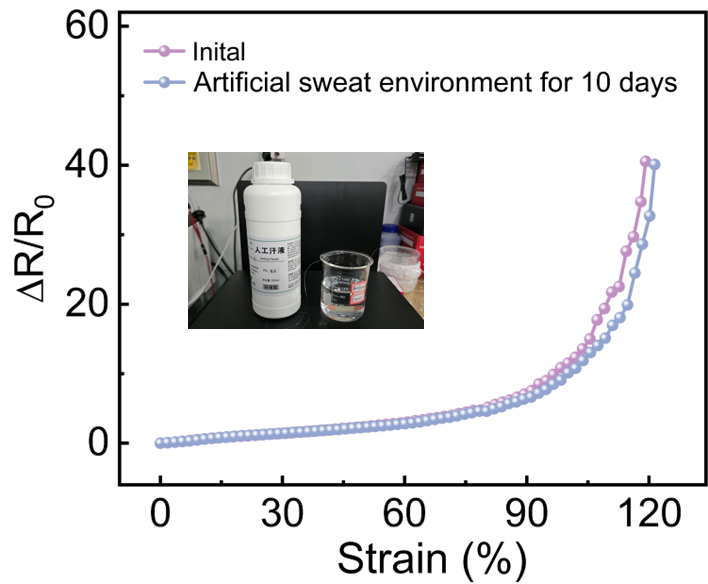


Fig. S31 The electromechanical performance of the metal film-based sensor before and after immersion in an artificial sweat solution for 10 days. The inset shows the environment in which the sensor was placed


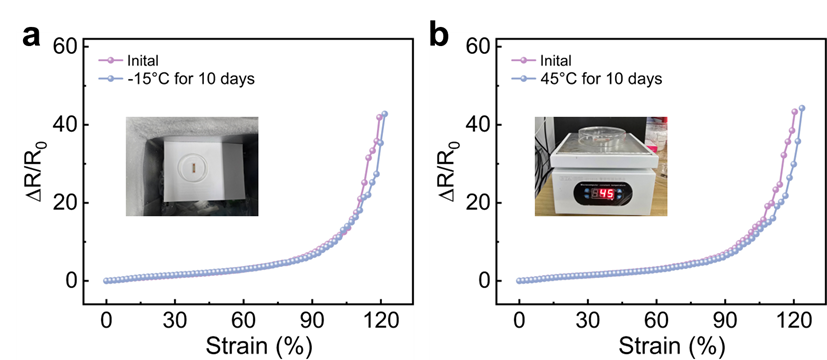


Fig. S32 The electromechanical performance of the metal film-based sensor before and after 10-day storage at (**a**) -15 °C and (**b**) 45 °C, respectively. The inset shows the environment in which the sensor was placed


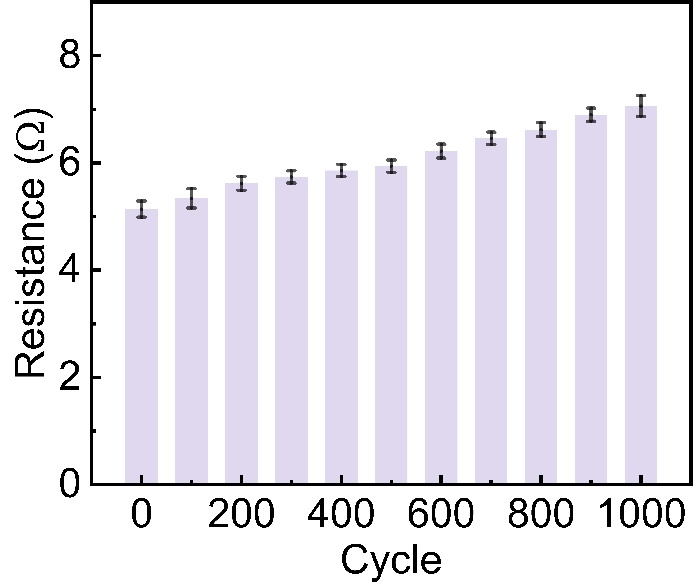


Fig. S33 The initial resistance of the strain-insensitive and stretchable metal films after the repeated cyclic testing under 150% strain


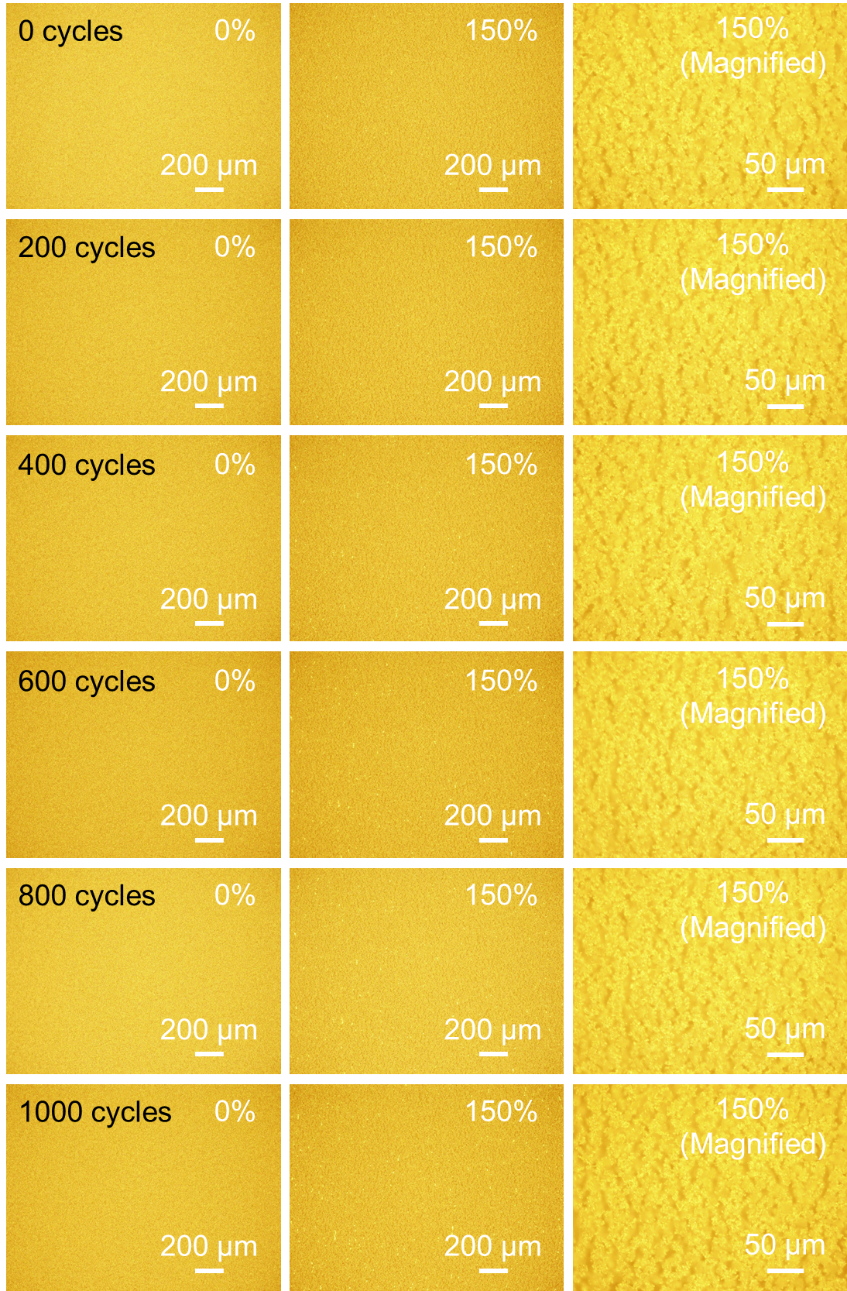


Fig. S34 The surface morphology of the strain-insensitive and stretchable metal films before and after cyclic testing. The last column shows the 5-folds higher magnification images (50x microscope) of samples


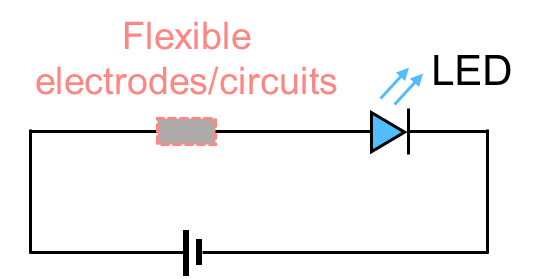


Fig. S35 Schematic of a closed loop circuit in which the metal films serving flexible electrodes/circuits are connected in series with a commercial light-emitting diode (LED) under a source voltage of 3 V


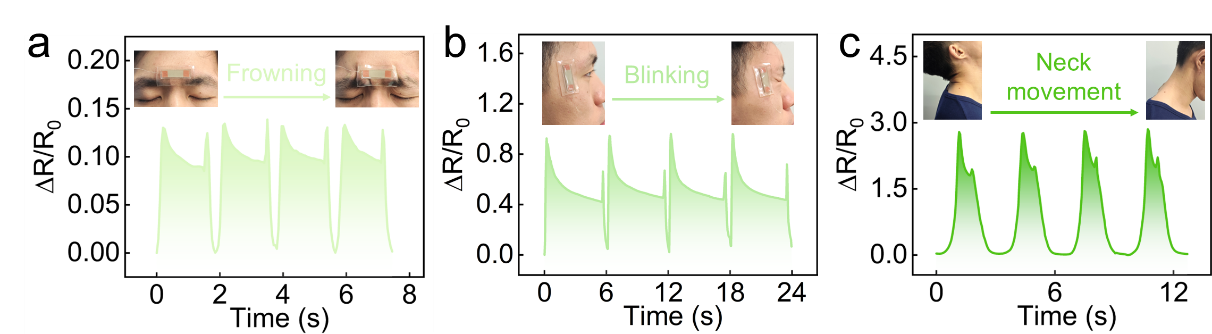


Fig. S36 Facial activity monitoring: real-time resistance signals during frowning, blinking, and neck movement


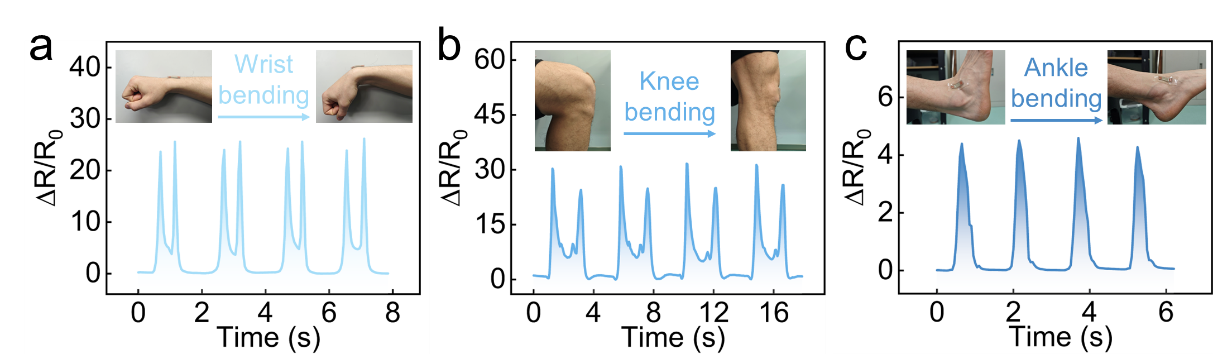


Fig. S37 Body motion sensing: real-time resistance signals during wrist, knee, and ankle bending


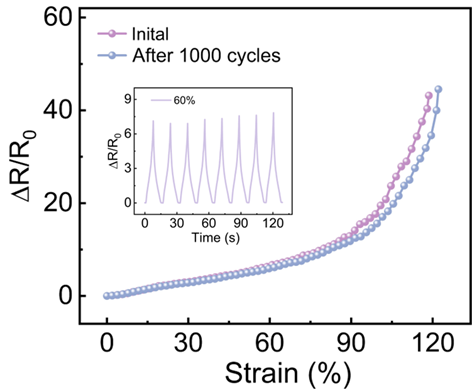


Fig. S38 The electromechanical performance of the metal film-based sensor before and after repeated cyclic loading (loading strain: 60%, loading speed: 0.5 mm s^-1^). The inset shows the loading conditions for the cyclic tests


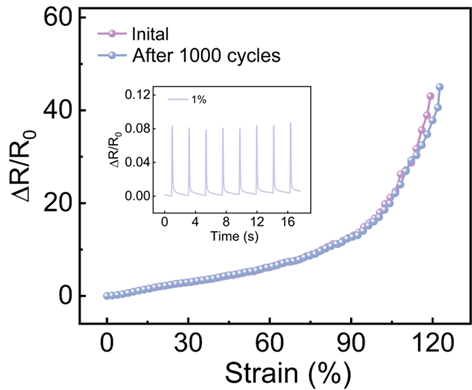


Fig. S39 The electromechanical performance of the metal film-based sensor before and after repeated cyclic loading (loading strain: 1%, loading speed: 1.5 mm s^-1^). The inset shows the loading conditions for the cyclic tests


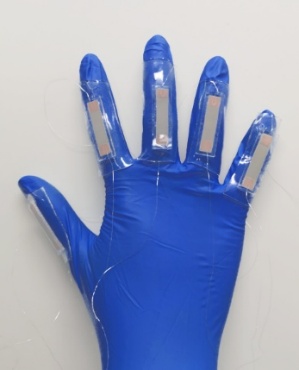


Fig. S40 Photograph of five independently sensors attached to each finger of a volunteer


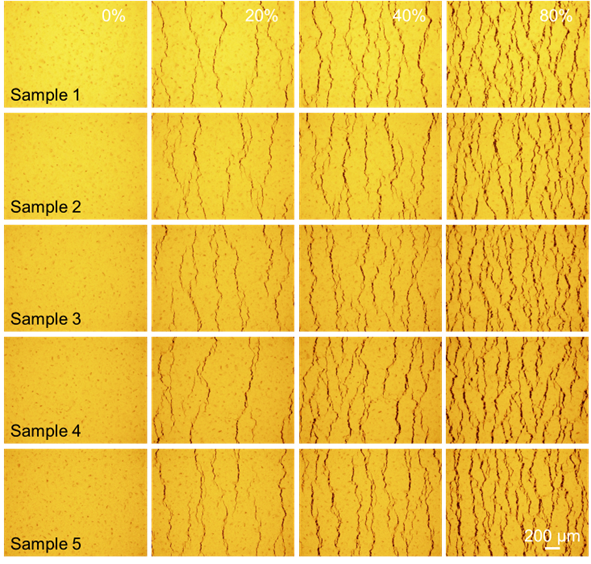


Fig. S41 The strain-dependent morphology evolution of five randomly selected samples


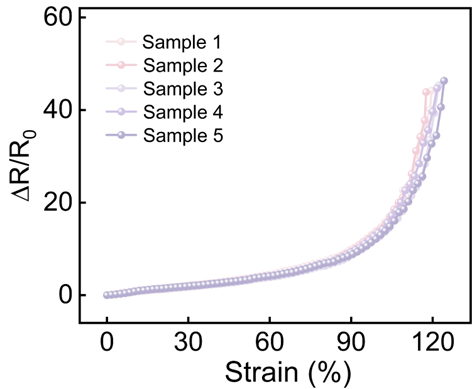


Fig. S42 The electromechanical performance of five sensor samples


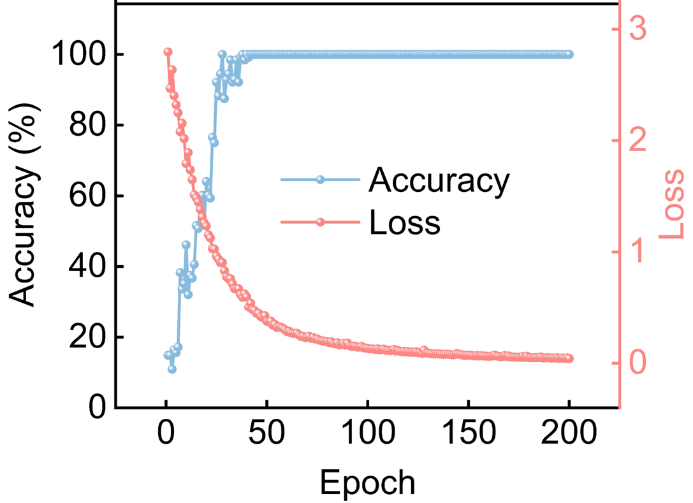


Fig. S43 Evolution of recognition accuracy and loss on the held-out test set over 200 training epochs for sign gesture translation. No early stopping or additional regularization was applied in this proof-of-concept study


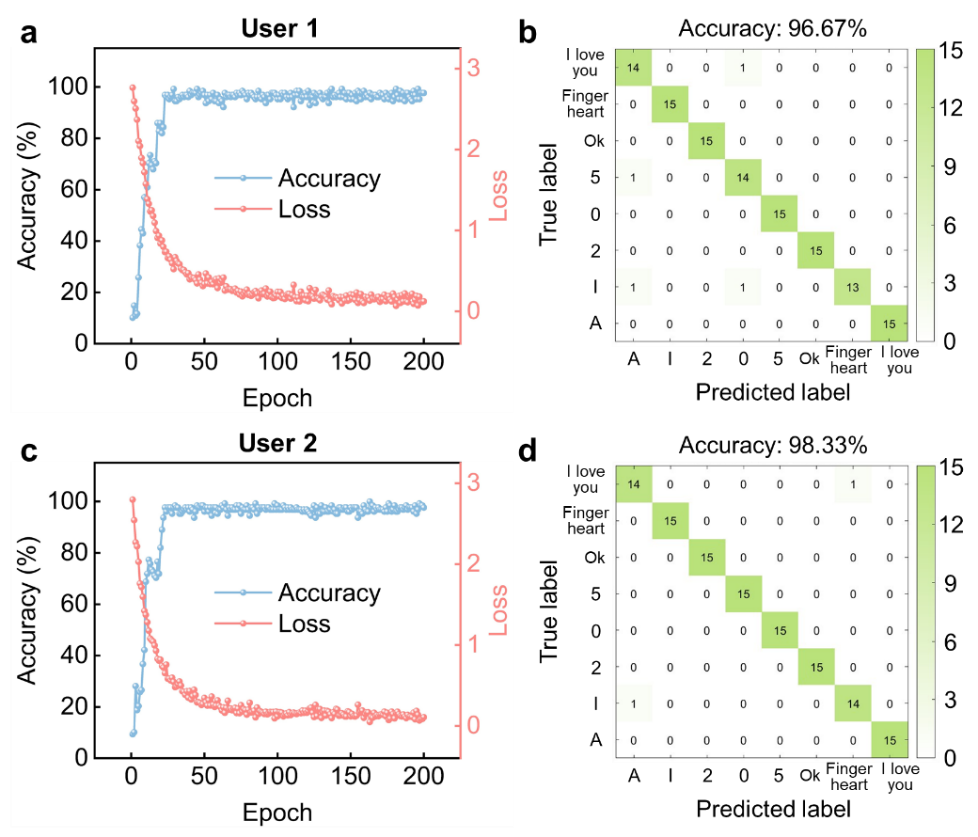


Fig. S44 The validation of the sign language translation model by multiple users. (**a**) Confusion matrix and (**b**) Evolution of recognition accuracy and loss on the held-out test set over training epochs for User 1. (**c**) Confusion matrix and (**d**) Evolution of recognition accuracy and loss on the held-out test set over training epochs for User 2


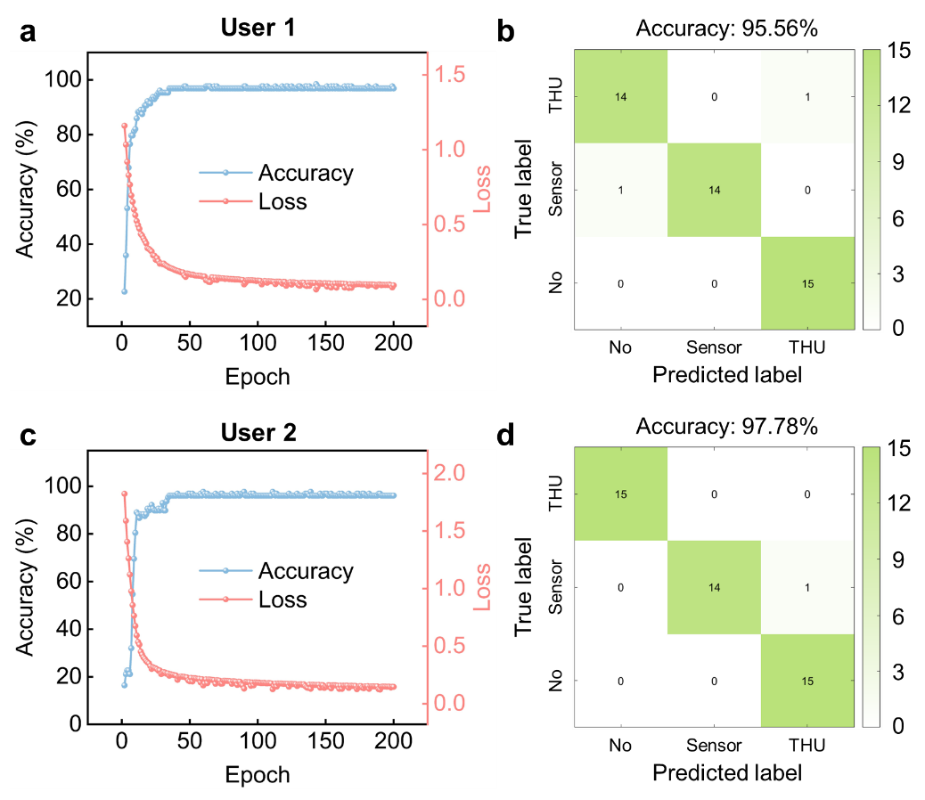


Fig. S45 The validation of the speech recognition model by multiple users. (**a**) Confusion matrix and (**b**) Evolution of recognition accuracy and loss on the held-out test set over training epochs for User 1. (**c**) Confusion matrix and (**d**) Evolution of recognition accuracy and loss on the held-out test set over training epochs for User 2

Supplementary Tables

Table S1. The performance comparison of the proposed dual-scale crack-manipulated metal film with previously deposited metal counterparts.

| Materials | Fabrication method | Design strategy | Maximum stretchability  (ɛ_max_) | ΔR/R_0_ at ɛ_max_ | Stretchability-tunable range | Sensitivity  (overall GF） | Response/  recovery time (ms) | Durability  (cycle number) | References |
| --- | --- | --- | --- | --- | --- | --- | --- | --- | --- |
| **Ag/PDMS** | **Pulsed laser deposition** | **Micro-nano, dual-scale crack manipulation** | **325%** | **3.51** | **0~325%** | **1.1** | **50/70** | **800** | **This work** |
| Pt/PUA | Sputtering | Spider-inspired nanoscale crack junction | 2% | 45 | 2% | 2250 | ~ | 5000 | [S18] |
| PET/Ag/PDMS | Printing | laminated structure | 40% | 10 | 40% | 25 | ~ | 5000 | [S19] |
| Au/PU | Sputtering | Micro/nano bulge structure | 80% | 16 | 80% | 20 | ~ | 1000 | [S20] |
| Au/PDMS | Reactive ion etching (RIE), DC sputter system | Nanosphere lithography-based strategy | 94% | 140 | 94% | 148.9 | ~ | 1000 | [S21] |
| Pt/AgNW/DS composite | Spray coating and sputtering deposition technique | Geometrical modulation | 100% | 25 | 0~100% | 25 | 205 | 1000 | [S22] |
| Au/PDMS | Thermal evaporation | 3D gyrified structure | 100% | 1.5 | 100% | 1.5 | ~ | 10000 | [S23] |
| Au/PDMS | Thermal evaporation | Surface chemistry modification | 120% | 34 | 0~120% | 28.3 | ~ | 1000 | [S24] |
| Au/SEBS | Magnetron sputtering | Hybrid Strategy | 120% | 15 | 120% | 12.5 | ~ | 1000 | [S25] |
| Ag/PDMS | Pulsed laser deposition | One-step defect-implantation | 200% | 6.2 | 0~200% | 3.1 | 50/75 | 500 | [S26] |
| Au/PDMS_0.9_-IPDI | Thermal evaporation | Double-microcrack coupling strategy | 200% | 200 | 125~200% | 100 | ~ | 1000 | [S27] |
| Ag-FeO_X_/PDMS | Pulsed laser deposition | Interlayer regulation strategy | 295% | 3.85 | 0~295% | 1.3 | 55/75 | 1000 | [S28] |
| Au/PDMS | Thermal evaporation | Substrate thermal expansion | 300% | 80 | 300% | 26.7 | ~ | 5000 | [S29] |

**Note:** Ranges were listed when the sensing performance is tunable; otherwise, single maximum values are listed.

Table S2 The performance comparison of the proposed dual-scale crack-manipulated metal film with liquid metals, conductive polymers, and nanocomposite-based conductors

| Materials | Fabrication method | Design strategy | Maximum stretchability  (ɛ_max_) | ΔR/R_0_ at ɛ_max_ | Stretchability-tunable range | Sensitivity  (overall GF） | Response/  recovery time (ms) | Durability  (cycle number) | References |
| --- | --- | --- | --- | --- | --- | --- | --- | --- | --- |
| **Ag/PDMS** | **Pulsed laser deposition** | **Micro-nano, dual-scale crack manipulation** | **325%** | **3.51** | **0~325%** | **1.1** | **50/70** | **800** | **This work** |
| Ga-In alloys | Plastic deformation | Phase transition | 110% | 3.41 | ~ | 3.1 | ~ | 7500 | [S6] |
| Liquid metal microgranular-particles | Meniscus-guided printing | ~ | 500% | 0.33 | ~ | 0.066 | ~ | 10000 | [S13] |
| Biphasic liquid metal composite | Deposition, printing | Biphasic liquid metal particle | 1200% | 1.4 | ~ | 0.11 | ~ | 5000 | [S17] |
| Liquid-metal fibre mat | Electrospinning, coating, printing | Substrate pre-stretching | 1800% | 0.041 | ~ | 0.002 | ~ | 10000 | [S12] |
| Liquid metal particle ink | Digital light processing-based projection lithography | ~ | 2500% | 44 | ~ | 1.76 | ~ | 1500 | [S15] |
| Ion-conductive elastomer | Ultraviolet-initiated polymerization | ~ | 266% | 3.4 | ~ | 1.276 | ~ | 1200 | [S30] |
| Cellulose-based conductive hydrogels | Autocatalytic fast polymerization strategy | ~ | 400% | 16 | ~ | 4 | 114/106 | 150 | [S31] |
| Cu-tannic @cellulose nanofibers/  glycerol hydrogels | Cu-tannic autocatalytic strategy | ~ | 400% | 15 | ~ | 3.75 | 120 | 200 | [S32] |
| Poly(SBMA-co-AA)/Al3+ multicomponent hydrogel | Photoinitiated polymerization | ~ | 800% | 10 | ~ | 1.25 | ~ | 50 | [S33] |
| GNP/MWCNT/  silicone rubber | Layer-by-layer assembly | Serpentine-shaped sensing layer | 100% | 2800 | ~ | 2800 | 46 | 2000 | [S34] |
| MWCNT/WPU yarn | Wet-spinning | Serpentine structure | 100% | 0.016 | ~ | 0.016 | ~ | 1000 | [S35] |
| CNTs ink/PU yarn | Swelling and sonication process | Wrinkle-assisted crack microstructure | 200% | 1250 | ~ | 625 | 88 | 1000 | [S36] |
| Nanocomposite organogel | Free radical polymerization | Surface modification | 650% | 10.5 | ~ | 1.6 | 70/70 | 2000 | [S37] |

**Note:** Ranges were listed when the sensing performance is tunable; otherwise, single maximum values are listed.

**Table S3** From structural design to application: Linking dual-scale crack manipulation strategy to tunable electromechanical performance and device-level functions

| Structural design | Crack patterns | Working ranges | Device-level functions |
| --- | --- | --- | --- |
| Compact metal films deposited on smooth substrates (without crack manipulation) | Through-film cracks | ~12% | Temperature sensors |
| Compact metal films deposited on microbump-roughened substrates (microscale crack manipulation) | Winding cracks | 75~145% | Wide-range strain sensors (human motion monitoring, sign language translation, and speech recognition) |
| Nanopore-implanted metal films deposited on microbump-roughened substrates (dual-scale crack manipulation) | Tiny-networked cracks | 225~325% | Stretchable electrodes/circuits (LED circuit and smartphone charging) |

Supplementary References

1. O.V. Devitsky, Influence of *Argon* pressure on the surface morphology of thin InGaAsP/Si films. The Viii International Young Researchers’ Conference – Physics, Technology, Innovations (Pti-2021) Ekaterinburg, Russia. AIP Publishing, (2022).: 030002. <https://doi.org/10.1063/5.0088641>
2. J.-P. Fang, J. Cai, Q. Wang, K. Zheng, Y.-K. Zhou et al., Low temperature Au-Au bonding using Ag nanoparticles as intermediate for die attachment in power device packaging. Appl. Surf. Sci. **593**, 153436 (2022). <https://doi.org/10.1016/j.apsusc.2022.153436>
3. M. Castillejo, P.M. Ossi, L. Zhigilei, Lasers in Materials Science (Springer International Publishing, 2014), <https://doi.org/10.1007/978-3-319-02898-9>
4. Y. Liu, Z. Xu, X. Ji, X. Xu, F. Chen et al., Ag-thiolate interactions to enable an ultrasensitive and stretchable MXene strain sensor with high temporospatial resolution. Nat. Commun. **15**(1), 5354 (2024). <https://doi.org/10.1038/s41467-024-49787-9>
5. J.-H. Lee, Y.-N. Kim, J. Lee, J. Jeon, J.-Y. Bae et al., Hypersensitive meta-crack strain sensor for real-time biomedical monitoring. Sci. Adv. **10**(51), eads9258 (2024). <https://doi.org/10.1126/sciadv.ads9258>
6. G. Li, M. Zhang, S. Liu, M. Yuan, J. Wu et al., Three-dimensional flexible electronics using solidified liquid metal with regulated plasticity. Nat. Electron. **6**(2), 154–163 (2023). <https://doi.org/10.1038/s41928-022-00914-8>
7. X.-W. Zhang, Y. Pan, Q. Zheng, X.-S. Yi, Time dependence of piezoresistance for the conductor-filled polymer composites. J. Polym. Sci. Part B Polym. Phys. **38**(21), 2739–2749 (2000). <https://doi.org/10.1002/1099-0488(20001101)38:21&lt;2739::AID-POLB40&gt;3.0.CO;2-O>
8. J. Zhao, G. Wang, R. Yang, X. Lu, M. Cheng et al., Tunable piezoresistivity of nanographene films for strain sensing. ACS Nano **9**(2), 1622–1629 (2015). <https://doi.org/10.1021/nn506341u>
9. M. Amjadi, A. Pichitpajongkit, S. Lee, S. Ryu, I. Park, Highly stretchable and sensitive strain sensor based on silver nanowire–elastomer nanocomposite. ACS Nano **8**(5), 5154–5163 (2014). <https://doi.org/10.1021/nn501204t>
10. S. Kang, M. Naqi, J. Shin, S. Lee, M. Lee et al., Laser-processed stretchable-gradient interconnection-based temperature sensor for a real-time monitoring system. ACS Appl. Electron. Mater. **3**(12), 5601–5607 (2021). <https://doi.org/10.1021/acsaelm.1c01052>
11. W. Yan, H. Li, J. Liu, J. Guo, EPMA and XRD study on nickel metal thin film for temperature sensor. Sens. Actuat. A Phys. **136**(1), 212–215 (2007). <https://doi.org/10.1016/j.sna.2006.11.012>
12. Z. Ma, Q. Huang, Q. Xu, Q. Zhuang, X. Zhao et al., Permeable superelastic liquid-metal fibre mat enables biocompatible and monolithic stretchable electronics. Nat. Mater. **20**(6), 859–868 (2021). <https://doi.org/10.1038/s41563-020-00902-3>
13. G.-H. Lee, Y.R. Lee, H. Kim, D.A. Kwon, H. Kim et al., Rapid *Meniscus*-guided printing of stable semi-solid-state liquid metal microgranular-particle for soft electronics. Nat. Commun. **13**, 2643 (2022). <https://doi.org/10.1038/s41467-022-30427-z>
14. S. Wang, Y. Nie, H. Zhu, Y. Xu, S. Cao et al., Intrinsically stretchable electronics with ultrahigh deformability to monitor dynamically moving organs. Sci. Adv. **8**(13), eabl5511 (2022). <https://doi.org/10.1126/sciadv.abl5511>
15. D. Wu, S. Wu, P. Narongdej, S. Duan, C. Chen et al., Fast and facile liquid metal printing *via* projection lithography for highly stretchable electronic circuits. Adv. Mater. **36**(34), 2307632 (2024). <https://doi.org/10.1002/adma.202307632>
16. Y. Xu, Y. Su, X. Xu, B. Arends, G. Zhao et al., Porous liquid metal-elastomer composites with high leakage resistance and antimicrobial property for skin-interfaced bioelectronics. Sci. Adv. **9**(1), eadf0575 (2023). <https://doi.org/10.1126/sciadv.adf0575>
17. D.H. Lee, T. Lim, J. Pyeon, H. Park, S.-W. Lee et al., Self-mixed biphasic liquid metal composite with ultra-high stretchability and strain-insensitivity for neuromorphic circuits. Adv. Mater. **36**(16), 2310956 (2024). <https://doi.org/10.1002/adma.202310956>
18. D. Kang, P.V. Pikhitsa, Y.W. Choi, C. Lee, S.S. Shin et al., Ultrasensitive mechanical crack-based sensor inspired by the spider sensory system. Nature **516**(7530), 222–226 (2014). <https://doi.org/10.1038/nature14002>
19. Z. Zheng, Z. Huang, N. Zhang, S. Liu, L. Zhao et al., Stretch-tolerant interconnects derived from silanization-assisted capping layer lamination for smart skin-attachable electronics. Mater. Today Phys. **46**, 101494 (2024). <https://doi.org/10.1016/j.mtphys.2024.101494>
20. J. Shu, R. Yang, Y. Chang, X. Guo, X. Yang, A flexible metal thin film strain sensor with micro/nano structure for large deformation and high sensitivity strain measurement. J. Alloys Compd. **879**, 160466 (2021). <https://doi.org/10.1016/j.jallcom.2021.160466>
21. Y. Ling, Q. Lyu, Q. Zhai, B. Zhu, S. Gong et al., Design of stretchable holey gold biosensing electrode for real-time cell monitoring. ACS Sens. **5**(10), 3165–3171 (2020). <https://doi.org/10.1021/acssensors.0c01297>
22. J.-Y. Noh, S.-H. Ha, G.R. Jeon, J.-M. Kim, Geometrical and electrical modulation of cracked metal films based on metal nanowire/elastomer composites for high-performance wearable strain sensing. Compos. Sci. Technol. **230**, 109738 (2022). <https://doi.org/10.1016/j.compscitech.2022.109738>
23. S. Chae, W.J. Choi, L.J. Nebel, C.H. Cho, Q.A. Besford et al., Kinetically controlled metal-elastomer nanophases for environmentally resilient stretchable electronics. Nat. Commun. **15**(1), 3071 (2024). <https://doi.org/10.1038/s41467-024-47223-6>
24. J. Zhu, X. Wu, J. Jan, S. Du, J. Evans et al., Tuning strain sensor performance *via* programmed thin-film crack evolution. ACS Appl. Mater. Interfaces **13**(32), 38105–38113 (2021). <https://doi.org/10.1021/acsami.1c10975>
25. H. Li, F. Han, L. Wang, L. Huang, O.W. Samuel et al., A hybrid strategy-based ultra-narrow stretchable microelectrodes with cell-level resolution. Adv. Funct. Mater. **33**(29), 2300859 (2023). <https://doi.org/10.1002/adfm.202300859>
26. B. Feng, T. Sun, W. Wang, Y. Xiao, J. Huo et al., Venation-mimicking, ultrastretchable, room-temperature-attachable metal tapes for integrated electronic skins. Adv. Mater. **35**(8), 2208568 (2023). <https://doi.org/10.1002/adma.202208568>
27. D. Yang, G. Tian, C. Liang, Z. Yang, Q. Zhao et al., Double-microcrack coupling stretchable neural electrode for electrophysiological communication. Adv. Funct. Mater. **33**(37), 2300412 (2023). <https://doi.org/10.1002/adfm.202300412>
28. T. Sun, B. Feng, J. Huo, Y. Xiao, J. Peng et al., Switching ultra-stretchability and sensitivity in metal films for electronic skins: a pufferfish-inspired, interlayer regulation strategy. Mater. Horiz. **10**(7), 2525–2534 (2023). <https://doi.org/10.1039/D3MH00252G>
29. Z. Jiang, N. Chen, Z. Yi, J. Zhong, F. Zhang et al., A 1.3-micrometre-thick elastic conductor for seamless on-skin and implantable sensors. Nat. Electron. **5**(11), 784–793 (2022). <https://doi.org/10.1038/s41928-022-00868-x>
30. Z. Cui, B. Jiang, X. Dai, Z. Guo, J. Niu et al., Self-adhesive, mechanically strong, and conductive organogel for flexible/wearable electronics and underwater electronic skin. Chem. Eng. J. **500**, 157551 (2024). <https://doi.org/10.1016/j.cej.2024.157551>
31. S. Zong, X. Wen, F. Lei, L. Zhu, J. Jiang et al., Construction of environmentally stable self-adhesive conductive cellulose hydrogel for electronic skin sensor *via* autocatalytic fast polymerization strategy at room temperature. Int. J. Biol. Macromol. **298**, 139999 (2025). <https://doi.org/10.1016/j.ijbiomac.2025.139999>
32. S. Zong, H. Lv, C. Liu, L. Zhu, J. Duan et al., Mussel inspired Cu-tannic autocatalytic strategy for rapid self-polymerization of conductive and adhesive hydrogel sensors with extreme environmental tolerance. Chem. Eng. J. **465**, 142831 (2023). <https://doi.org/10.1016/j.cej.2023.142831>
33. P. Zhang, Y. Zhang, X. Yu, K. Liu, M. Wang et al., A multifunctional ionic-conductive hydrogel sensor with zwitterionic polymer/Al^3+^ crosslinking for wearable and underwater motion monitoring. Chem. Eng. J. **524**, 169618 (2025). <https://doi.org/10.1016/j.cej.2025.169618>
34. J. Zhou, X. Guo, Z. Xu, Q. Wu, J. Chen et al., Highly sensitive and stretchable strain sensors based on serpentine-shaped composite films for flexible electronic skin applications. Compos. Sci. Technol. **197**, 108215 (2020). <https://doi.org/10.1016/j.compscitech.2020.108215>
35. H. Yuan, R. Jia, H. Yao, W. Wang, K. Qian et al., Ultra-stable, waterproof and self-healing serpentine stretchable conductors based on WPU sheath-wrapped conductive yarn for stretchable interconnects and wearable heaters. Chem. Eng. J. **473**, 145251 (2023). <https://doi.org/10.1016/j.cej.2023.145251>
36. H. Sun, K. Dai, W. Zhai, Y. Zhou, J. Li et al., A highly sensitive and stretchable yarn strain sensor for human motion tracking utilizing a wrinkle-assisted crack structure. ACS Appl. Mater. Interfaces **11**(39), 36052–36062 (2019). <https://doi.org/10.1021/acsami.9b09229>
37. Y. Bai, Y.M. Chen, J. Tang, K. Li, Y. Zhang et al., Anti-fatigue, anti-puncture and self-recovery nanocomposite organogel-based strain sensor for trocar seal system in laparoscopic surgery. Chem. Eng. J. **518**, 164653 (2025). <https://doi.org/10.1016/j.cej.2025.164653>
